# The Landscape and Regulation of Histone Crotonylation in Mammalian Gametes and Early Embryos

**DOI:** 10.1002/advs.75143

**Published:** 2026-04-03

**Authors:** Shenli Yuan, Yelian Yan, Yalin Liang, Kanghua Zhong, Zhican Fu, Xiaobo Wang, Chao Liu, Tao Huang, Keliang Wu

**Affiliations:** ^1^ GMU‐GIBH Joint School of Life Sciences Guangzhou Women and Children's Medical Center Guangzhou Medical University Guangzhou Guangdong China; ^2^ State Key Laboratory of Reproductive Medicine and Offspring Health Center for Reproductive Medicine Institute of Women Children and Reproductive Health Shandong University Jinan Shandong China; ^3^ Beijing Life Science Academy Key Laboratory of Tobacco Biological Effects Beijing China

**Keywords:** Epigenetic reprogramming, Embryonic development, Histone crotonylation, ZGA

## Abstract

Histone crotonylation is a newly recognized epigenetic modification implicated in transcriptional regulation, cell‐fate determination, and disease. However, its dynamics and regulation during mammalian gametes and early embryonic development remain largely unknown. Here, we apply an optimized ULI‐NChIP‐seq to establish a high‐resolution landscape of H3K18 crotonylation (H3K18cr), a representative histone crotonylation mark, in mouse gametes and preimplantation embryos. We report that H3K18cr is enriched in sperm but largely absent in MII oocyte, and undergoes striking reprogramming after fertilization. Maternal‐biased parental asymmetry is strongest at the zygote stage and becomes balanced as development progresses. Paternal‐ and maternal‐specific H3K18cr occupy distinct genomic regions with divergent biological functions. Interestingly, H3K18cr undergoes a global transition from broad domains to narrow peaks at minor ZGA stage. This reprogramming is strictly dependent on minor ZGA, but essential for major ZGA and embryonic development. Inhibiting histone deacetylase activity of the HDAC1 complex not only enhances histone acetylation signal, but also impairs the broad‐to‐narrow transition of histone crotonylation, revealing functional cross‐talk between histone crotonylation and acetylation. Following reprogramming, canonical narrow H3K18cr signals can potentially influence gene expression, first‐lineage differentiation, and blastocyst development. Collectively, histone crotonylation undergoes extensive reprogramming during early embryogenesis, and its interplay with ZGA links epigenetic regulation to development.

## Graphical abstract

Histone crotonylation undergoes a genome‐wide transition from broad domains to canonical narrow peaks during minor zygotic genome activation (ZGA). This remodeling is required for proper major ZGA and blastocyst formation. Disruption of this transition by transcriptional inhibition, metabolic perturbation, or HDAC1 dysfunction impairs embryonic transcriptional competence.

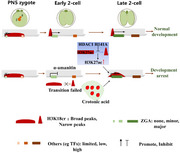

## 1 Introduction

Epigenetic regulation is fundamental to mammalian reproduction and development, coordinating chromatin organization and gene expression programs. Previous studies have focused primarily on classical epigenetic modifications such as DNA methylation, histone methylation, and acetylation. Histone lysine crotonylation (Kcr), first reported in 2011 as a crotonyl‐CoA–dependent post‐translational modification on lysine residues, has since emerged as a novel epigenetic mark with critical roles in transcriptional regulation, cell fate determination, and disease pathogenesis [1, 2, 3, 4, 5]. Its unique chemical structure distinguishes it from acetylation and enables important functions in transcriptional regulation, chromatin architecture, and diverse biological processes [1, 6, 7, 8, 9]. The homeostasis of Kcr is maintained through the coordinated action of “writers” (e.g., p300/CBP, MOF), “erasers” (SIRT1–3, HDAC1–3), and “readers” (YEATS‐domain proteins) [10, 11, 12, 13, 14, 15], with p300‐mediated crotonylation showing particularly strong transcriptional activation potential [10, 11]. Kcr is widely enriched at promoters and enhancers [5] and has been implicated in embryonic stem cell self‐renewal [13], spermatogenesis [16], and embryonic development [1].

It is also tightly associated with disease, as evidenced by crotonate supplementation elevating Kcr to protect renal function in acute injury [7], ECHS1 regulating H3K18cr to sustain cardiac metabolic homeostasis [17], and aberrant elevation of H3K27cr correlating with colorectal cancer progression [18]. These findings establish Kcr as a unique and irreplaceable epigenetic modification in both development and disease.

Mammalian preimplantation development is a highly dynamic and tightly regulated process encompassing zygotic genome activation (ZGA), compaction, and the first lineage segregation [19]. ZGA represents the pivotal transition from maternal control to embryonic transcription. In mice, minor and major ZGA occur sequentially at the early and late two‐cell stages, whereas in humans, ZGA is initiated mainly at the eight‐cell stage [19, 20]. This process is driven by maternal factor dilution, activator accumulation, and chromatin remodeling, and involves key transcription factors such as OCT4, NFYA, DUX, OBOX, and KLF families [19, 21]. Epigenetic reprogramming is a hallmark of mammalian preimplantation development [21], involving the resetting of DNA methylation and histone modifications such as H3K4me3 and H3K27me3, which collectively re‐establish totipotency, enable zygotic genome activation, and safeguard normal embryogenesis [20, 22].

For example, global DNA methylation levels decline rapidly after fertilization, reach their lowest point at the blastocyst stage, and then gradually recover [22, 23]. The paternal genome undergoes active demethylation via TET3, whereas the maternal genome is primarily demethylated passively, with protection from maternal factors such as STELLA and UHRF1 [24, 25, 26, 27]. Despite widespread demethylation, differentially methylated regions at imprinted genes are preserved to ensure stable inheritance [23]. Histone modifications also undergo dynamic reprogramming. For example, H3K4me3 is initially present as broad domains in oocytes but is reset to canonical narrow peaks at the major ZGA stage, a transition that facilitates gene activation [28, 29, 30]. In contrast, H3K27me3 is rapidly erased from the paternal genome but partially retained maternally, where it contributes to the regulation of early embryonic transcription [31, 32]. Collectively, these studies highlight the central roles of classical epigenetic modifications in restoring totipotency and activating the embryonic genome [21, 33]. However, compared with DNA methylation and classical histone modifications, the fine‐tuned dynamic reprogramming of novel epigenetic marks such as Kcr in gametes and early embryos remains largely unexplored, particularly with respect to their regulation and their interplay with ZGA and lineage specification. Systematic investigation of Kcr dynamics during fertilization and preimplantation development will provide new insights into the fine epigenetic control of early embryogenesis, which could offer potential avenues for improving reproductive health and developing strategies for the diagnosis and treatment of developmental disorders.

## 2 Results

### 2.1 Dynamics of H3K18cr in Mouse Gametes and Early Embryos

**FIGURE 1:**
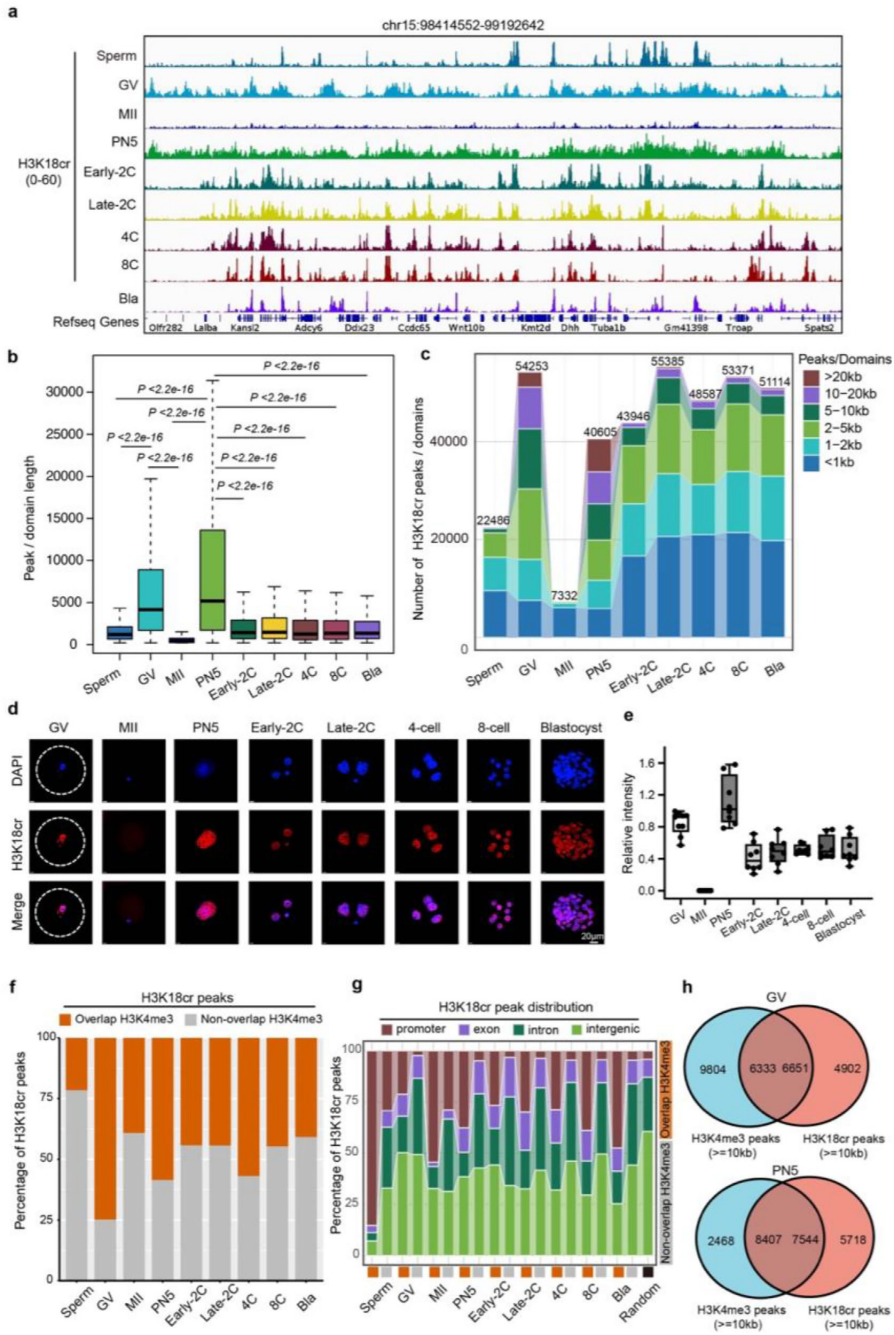
Dynamic landscape of H3K18cr in mouse gametes and early embryos. (a) Genome browser view of H3K18cr enrichment in sperm, GV oocyte, MII oocyte, and preimplantation embryos. 2C represents two‐cell embryo; 4C represents four‐cell embryo; 8C represents an eight‐cell embryo; Bla represents a blastocyst. (b) Box plots comparing the lengths of H3K18cr peaks (or domains) among the gametes and embryos. The Wilcoxon rank sum test was used. (c) The number of H3K18cr peaks or domains within different ranges of lengths in the gametes and embryos. (d) Immunostaining of H3K18cr in mouse germinal vesicle (GV) oocytes (n = 9), metaphase II (MII) oocytes (n = 8), zygotes (n = 8), early two‐cell (n = 8), late two‐cell (n = 8), four‐cell(n = 8), eight‐cell(n = 8) and blastocyst (n = 8) embryos. DNA is stained by DAPI (blue). H3K18cr signal is shown in red color. Scale bar, 20 µm. (e) Box plot showing the quantification of relative signal intensity of H3K18cr immunostaining at different stages. (f) Percentage of H3K18cr peaks that overlap or do not overlap with H3K4me3 peaks at the indicated stages in mouse gametes and early embryos. (g) Bar plot comparing the distribution of H3K18cr peaks that overlap or do not overlap with H3K4me3 peaks across genomic elements. (h) Venn diagrams showing the overlap between H3K4me3 and H3K18cr broad peaks (≥10 kb) in GV oocytes and PN5 zygotes. For example, in the overlapping region, 6333 and 6651 represent the number of overlapping broad peaks for H3K4me3 and H3K18cr, respectively, in GV oocytes.

To investigate the dynamic reprogramming and regulation of Kcr in gametes and early embryos, we applied an optimized ultra‐low‐input native chromatin immunoprecipitation sequencing (ULI‐NChIP‐seq) method, suitable for limited cell numbers [29, 34, 35], to generate genome‐wide H3K18cr maps from gametes to E3.5 blastocysts (Figure S1a and Table S1). For each developmental stage, at least two biological replicates were included. Our data indicated that all biological replicates at each developmental stage showed high reproducibility (Figure S1b). Through systematic analysis of gametes and early embryos, we found that H3K18cr undergoes marked reprogramming after fertilization (Figure 1a). Pronounced H3K18cr signals were detected in germinal vesicle (GV) oocyte and sperm, whereas the modification was nearly absent in metaphase II (MII) oocyte (Figure 1a,b). Following fertilization, H3K18cr signals were dramatically reestablished in zygotes (Figure 1a). More than 40,000 H3K18cr peaks were identified at this stage, and their average length was markedly greater than those in MII oocyte or sperm (Figure 1b,c). Immunofluorescence staining further supported this dynamic pattern (Figure 1d). H3K18cr signals were weak in MII oocytes but markedly increased in zygotes or early two‐cell embryos, followed by moderate levels in later stages, consistent with quantitative intensity measurements (Figure 1e). These results suggest a close link between re‐establishment of H3K18cr and fertilization. Interestingly, 21.3% (11553 / 54253) of H3K18cr peaks in GV oocytes and 32.7% (13262 / 40605) in zygotes displayed broad‐domain patterns (≥10 kb), respectively (Figure 1c; Table S2). At the early two‐cell stage (minor ZGA), most of these broad peaks or domains were lost and replaced by canonical narrow peaks, which remained stable throughout subsequent embryonic stages (Figure 1a,c). This pattern differs from H3K4me3, which is present as broad domains in MII oocytes and remains so until the early two‐cell stage, when it is reset to canonical narrow peaks during the major ZGA (late two‐cell) stage [30]. These differences suggest that H3K18cr and H3K4me3 are reprogrammed by distinct mechanisms and may play non‐redundant roles in coordinating epigenetic transitions around zygotic genome activation.

Genome‐wide profiling revealed that H3K18cr peaks were broadly distributed across promoters at all developmental stages (Figure S1c). Notably, sperm displayed the highest fraction of promoter‐associated H3K18cr peaks among all stages. From the two‐cell to blastocyst stage, the number of promoter‐associated peaks gradually increased, and the signals became progressively concentrated around transcription start sites (TSSs) (Figure S1d). Promoters marked by H3K18cr exhibited higher CpG density than those lacking the modification (Figure S1d), suggesting that H3K18cr preferentially marks CpG‐rich regulatory regions. These observations indicate that H3K18cr selectively occupies promoter regions with transcriptional potential, implying an important role in gene regulation during preimplantation development. To further elucidate the reprogramming trajectory of H3K18cr from gametes to early embryos, we examined its dynamics at matched genomic loci across developmental stages (Figure S2a–c). Pronounced parental asymmetry was observed, with GV oocytes exhibiting more extensive H3K18cr domains (broad peaks) than sperm, and displaying a pattern similar to that of PN5 zygotes (Figure 1a,c). In mouse zygotes, a substantial proportion of genomic bins with broad H3K18cr peaks were mainly originated from GV oocytes (Figure S2b). From the zygote to the early two‐cell stage, approximately 84% genomic bins (1 kb) with broad H3K18cr peaks underwent extensive erasure, while most narrow H3K18cr peaks in early two‐cell were newly formed or derived from the contraction of the zygote's broad peaks (Figure S2a). This process coincided precisely with the minor ZGA stage, indicating that the distribution pattern of H3K18cr undergoes a remarkable transition during early embryonic genome activation in mice. In addition, the genomic elements associated with dynamically changing H3K18cr regions showed distinct distribution patterns (Figure S2d). Together, these results delineate a highly dynamic reprogramming landscape of H3K18cr in mouse gametes and preimplantation embryos, highlighting its stage‐specific remodeling and close association with ZGA.

### 2.2 Associations of H3K18cr With Histone Methylation and DNA Methylation

As a novel histone acylation modification, the chromatin context of H3K18cr and its associations with classical epigenetic modifications remain poorly characterized. We therefore compared the genomic distribution of H3K18cr with major histone methylation marks and DNA methylation in mouse gametes and early embryos (Figure S3a,b). Both H3K18cr and H3K4me3 transitioned from broad domains to narrow peaks after fertilization (Figure S3c,d), whereas H3K27me3 largely retained extensive broad domains from gametes to early embryos (Figure S3e). Further analysis showed prominent broad H3K4me3 domains in MII oocytes and early two‐cell embryos, whereas H3K18cr signals were weak in MII oocytes but displayed a pronounced narrow‐peak pattern in early two‐cell embryos. These findings suggest distinct reprogramming dynamics between active and repressive epigenetic modifications during early embryogenesis. Genome‐wide comparisons revealed that a substantial fraction of H3K18cr peaks overlapped with the active histone mark H3K4me3, whereas overlap with the repressive mark H3K27me3 was limited (Figure 1f; Figure S3f). We next examined the genomic distribution of H3K18cr peaks according to their overlap with H3K4me3. Peaks overlapping H3K4me3 were more frequently located in promoter regions, whereas non‐overlapping peaks were more frequently distributed within intronic and intergenic regions (Figure 1g). Notably, as embryonic development progresses, H3K18cr peaks overlapping H3K4me3 show an increasing tendency to localize to promoter regions. Broad H3K18cr regions (≥10 kb) also showed substantial overlap with H3K4me3 domains in GV oocytes and PN5 zygotes (Figure 1h). In GV oocytes, 57.6% (6651 / 11553) of broad H3K18cr regions overlapped with H3K4me3 domains, and 56.9% (7544 / 13262) overlapped in PN5 zygotes. These results further support the coordinated deposition of H3K18cr and H3K4me3 at large chromatin regions.

To further investigate the functional relevance of H3K18cr‐associated chromatin regions, we performed gene ontology (GO) analysis for genes associated with promoter peaks that either overlapped between H3K18cr and H3K4me3 or were marked by H3K4me3 alone. Promoters co‐marked by H3K18cr and H3K4me3 were strongly enriched for fundamental cellular processes across stages from sperm to early embryos, including RNA processing, histone modification, and mitotic chromosome segregation (Figure S4a). In contrast, promoters marked by H3K4me3 alone in early embryos were preferentially enriched for developmental pathways, such as cell fate determination and organ development (Figure S4a). Notably, in sperm, promoters marked by H3K4me3 alone showed specific enrichment for processes related to spermatogenesis and sperm morphogenesis, highlighting distinct functional features of this promoter class. To further assess the regulatory features of H3K18cr‐associated chromatin regions, transcription factor motif enrichment analysis was performed for H3K18cr peaks (Figure S4b). During early cleavage stages (two‐cell to eight‐cell), enriched motifs were largely comparable between H3K18cr peaks with or without H3K4me3 overlap. However, at the blastocyst stage, motifs for KLF family members (KLF1/4/5/6), OTX2, and TEAD were preferentially enriched in H3K18cr‐only regions (Figure S4b), suggesting stage‐specific regulatory features associated with H3K18cr independent of H3K4me3.

To examine the relationship between H3K18cr and other epigenetic features, we analyzed the genomic association of H3K18cr peaks with DNA methylation and transposable elements (TEs). Across mouse gametes and early embryonic stages, CpG methylation levels at H3K18cr peaks were significantly lower than those at random genomic regions (Figure S5a). Consistently, H3K18cr peaks were predominantly distributed in low‐ to intermediate‐methylation categories (0–0.5), whereas random regions were more frequently associated with highly methylated states (Figure S5b), indicating that H3K18cr preferentially localizes to relatively hypomethylated genomic environments. Given the reported roles of histone modifications in regulating transposable element (TE) activity during early development [30, 36], we examined the association between H3K18cr peaks and major TE families. The fraction of H3K18cr peaks overlapping each TE family showed stage‐dependent patterns. Alu elements represented the largest fraction, decreasing from 70.2% at the early two‐cell stage to 49.2% at the blastocyst stage (Figure S5c). L1 elements accounted for 25.4% in late two‐cell embryos and 18.2% in blastocysts, whereas ERVK elements increased from 17.4% in early two‐cell embryos to 19.0–19.6% at later stages. Enrichment analysis further revealed stage‐specific associations, with L1 most enriched at the late two‐cell stage and ERVK at the 8‐cell stage (Figure S5d). These results indicate that H3K18cr‐marked chromatin regions are generally associated with hypomethylated genomic contexts and display dynamic relationships with distinct TE families during early embryogenesis.

### 2.3 Parent‐of‐Origin Differences in Histone Crotonylation During Mouse Preimplantation Development

**FIGURE 2:**
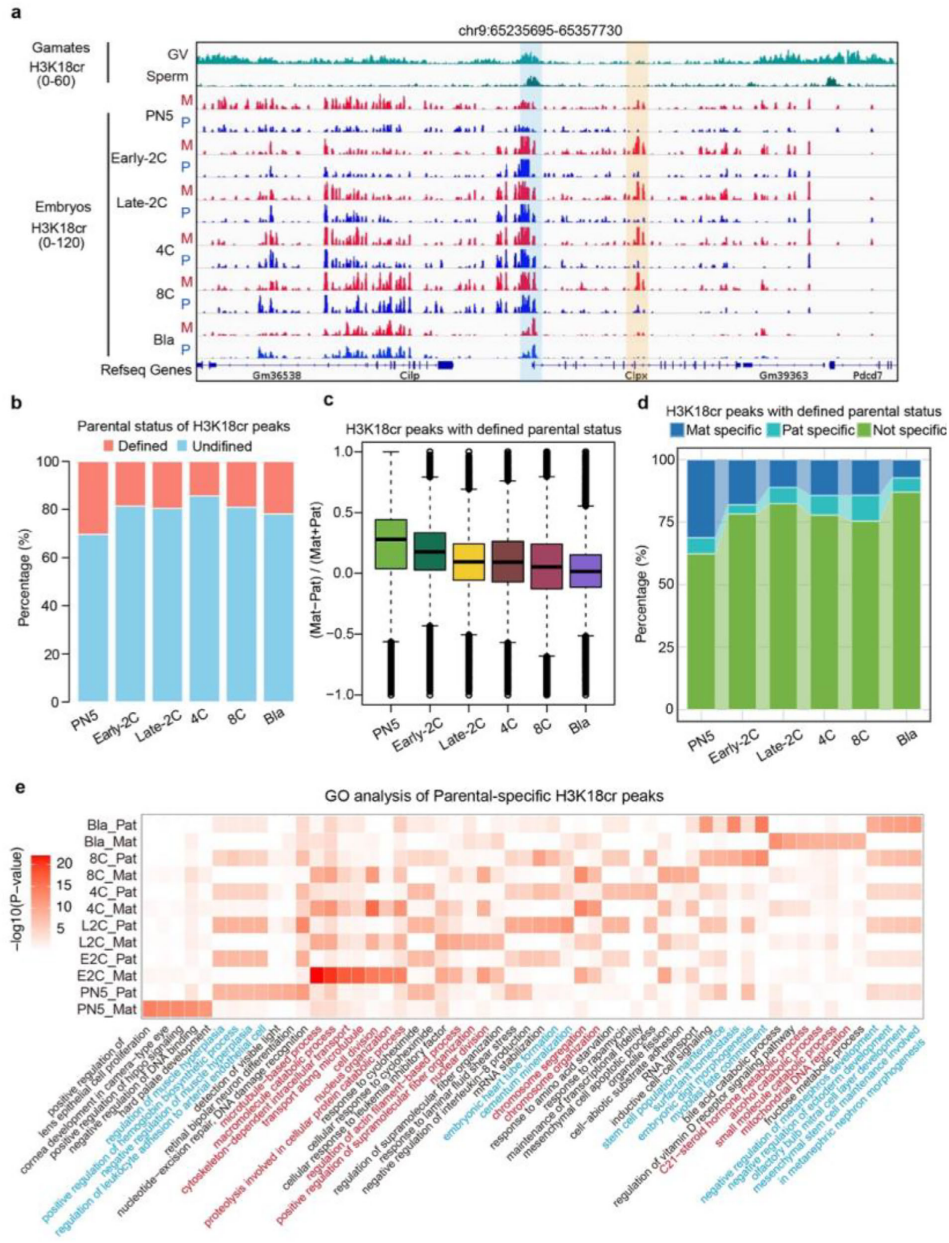
Differences and dynamic changes of H3K18cr between paternal and maternal genomes. (a) Genome browser views showing paternal and maternal H3K18cr signals in mouse gametes and embryos. (b) Bar plot showing the proportion of H3K18cr peaks with defined parental status, which were determined based on SNPs covered by sufficient effective reads. (c) Box plots comparing the differences in H3K18cr signal between the paternal and maternal genomes for peaks with defined parental status. (d) Bar plot showing the proportions of H3K18cr peaks with defined parental status that were classified as maternal‐specific (Mat specific), paternal‐specific (Pat specific), and non‐parental‐specific (Not specific). (e) GO analysis of parental‐specific H3K18cr peaks in different embryo stages.

Epigenetic asymmetry between the paternal and maternal genomes, such as genomic imprinting via DNA methylation or H3K27me3 [32, 36], is crucial for early embryonic development. To investigate parental differences in H3K18cr deposition, we analyzed hybrid mouse embryos using informative SNPs to distinguish the parental origin of H3K18cr reads. Pronounced asymmetry was observed between the two parental genomes (Figure 2a). At the PN5 zygote stage, the global difference in H3K18cr levels was maximal, with markedly stronger signals on the maternal genome (Figure 2b,c). As embryonic development progressed, the overall parental disparity in H3K18cr gradually diminished (Figure 2c). Analysis of parent‐specific H3K18cr peaks further revealed that maternal‐specific peaks dominated before and during minor ZGA, whereas the proportion of paternal‐ and maternal‐specific peaks became progressively balanced during and after major ZGA (Figure 2d).

Previous works have reported that distal regulatory elements, such as enhancers, also harbor histone crotonylation enrichment. By comparing parental‐specific and non‐specific peaks, we found that parental‐specific H3K18cr peaks were more frequently located in distal regions rather than promoters (Figure S5e). Functional annotation analysis identified distinct functional enrichments associated with paternal‐ and maternal‐specific H3K18cr regions. Paternal‐specific regions were enriched for genes involved in organ and tissue development, whereas maternal‐specific regions were preferentially associated with fundamental cellular processes (Figure 2e). These findings reveal that parental asymmetry of H3K18cr is not only reflected in its global intensity but also in its genomic distribution and potential regulatory function, suggesting distinct contributions of the maternal and paternal genomes to early embryonic epigenetic regulation.

### 2.4 Minor ZGA‐Dependent Transition of H3K18cr From Broad to Narrow Peaks in Early Mouse Embryos

**FIGURE 3:**
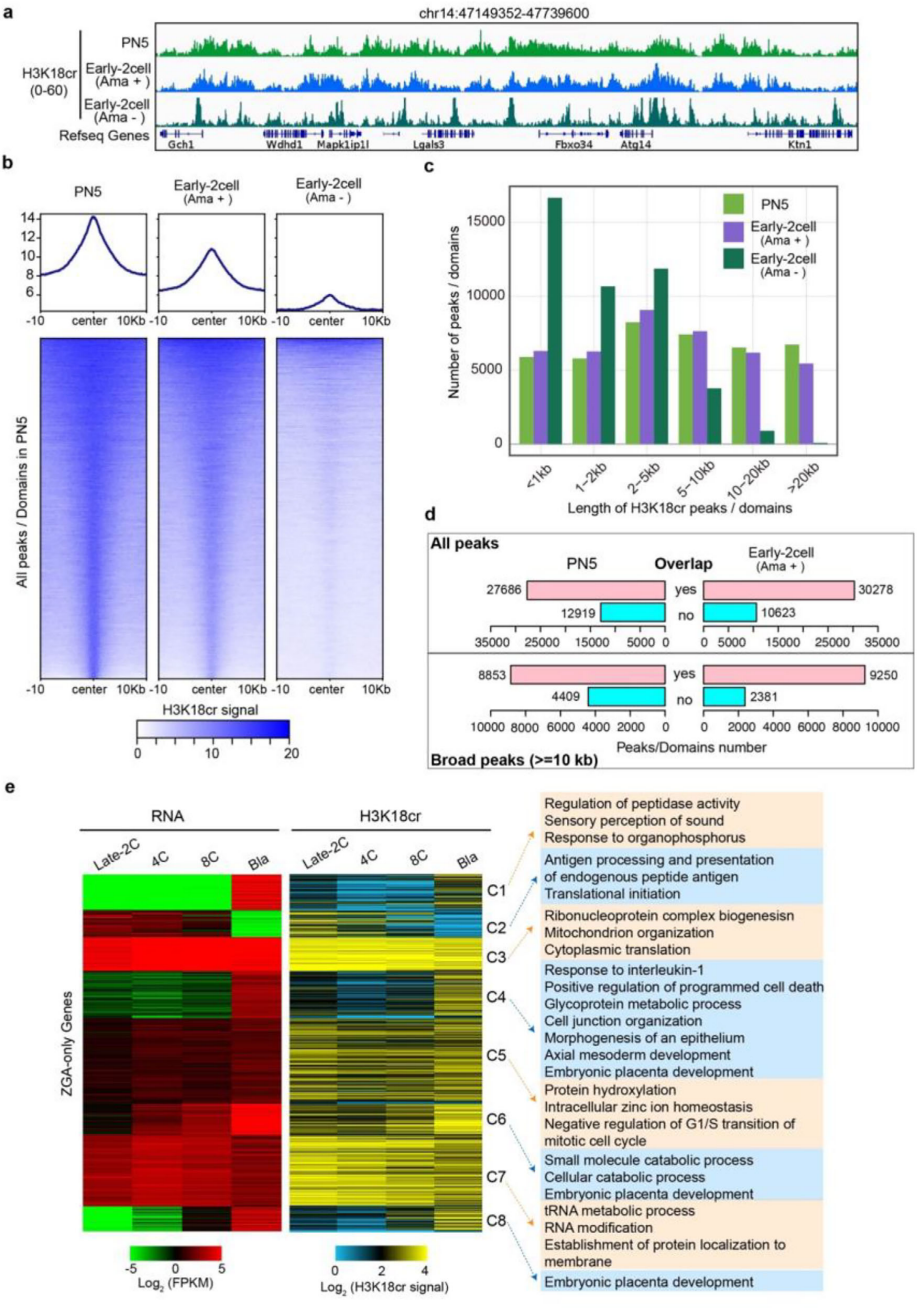
The transition of H3K18cr from broad to narrow peaks requires minor ZGA. (a) Genome browser view of H3K18cr signals in PN5 zygotes, early two‐cell embryos, and α‐amanitin‐treated early two‐cell embryos. (b) Heatmap showing changes in H3K18cr signals of zygote H3K18cr peaks in PN5 zygotes, early two‐cell embryos, and α‐amanitin‐treated early two‐cell embryos. (c) The number of H3K18cr peaks or domains with different ranges of lengths in mouse zygotes, early two‐cell embryos, and early two‐cell treated by α‐amanitin. (d) Bar plot showing the overlap of all H3K18cr peaks (top) or broad H3K18cr peaks (≥10 kb) (below) between PN5 zygotes and α‐amanitin‐treated early two‐cell embryos. (e) Heatmap showing the expression of ZGA‐only genes and H3K18cr signal enrichment at their promoters across early embryonic stages. Based on K‐means clustering, ZGA‐only genes were divided into 8 clusters (C1–C8). Gene Ontology (GO) enrichment analysis for each cluster was shown on the right side of the heatmap.

Our previous analyses revealed that broad H3K18cr domains were extensively erased at the minor ZGA stage, accompanied by the emergence of canonical narrow peaks. This observation suggested that the transition of H3K18cr from broad to narrow peaks might depend on minor ZGA. To verify this hypothesis, embryos were treated with α‐amanitin from the PN3 zygotic stage onward, a well‐characterized inhibitor of embryonic genome transcription [30, 37]. In early two‐cell embryos exposed to α‐amanitin, the global H3K18cr landscape remained highly similar to that of normal PN5 zygotes, exhibiting extensive broad‐domain distributions of H3K18cr signals (Figure 3a,b). In contrast, untreated embryos exhibited a genome‐wide transition to discrete, sharply localized H3K18cr peaks characteristic of canonical chromatin marking during the minor ZGA stage (Figure 3a). Further quantitative analysis revealed that α‐amanitin treatment markedly shifted the distribution of H3K18cr peak lengths toward longer domains, whereas untreated embryos showed a strong enrichment of narrow peaks (Figure 3c). Consistently, overlap analysis demonstrated that α‐amanitin‐treated embryos retained a greater fraction of H3K18cr peaks inherited from PN5 zygotes (Figure 3d). Among all peaks, 74.0% (30278 / 40901) in α‐amanitin‐treated early two‐cell embryos overlapped with those in normal PN5 zygotes (Figure 3d). A slightly higher proportion was observed for broad peaks in α‐amanitin‐treated early two‐cell embryos, with 79.5% (9250 / 11631) overlapping with PN5 broad peaks (Figure 3d). These results demonstrate that inhibition of zygotic transcription prevents the normal erasure of broad H3K18cr domains and disrupts their conversion into canonical narrow peaks. Together with the persistence of PN5‐like H3K18cr patterns in α‐amanitin‐treated embryos, these findings provide direct evidence that transcriptional activation during minor ZGA is indispensable for H3K18cr domain remodeling and the establishment of a mature chromatin landscape in early embryos.

### 2.5 Transition of H3K18cr Pattern Contributes to Major ZGA Gene Expression

From the two‐cell to blastocyst stages, H3K18cr peaks across the genome predominantly adopt canonical narrow patterns, a chromatin signature often linked to transcriptional activation. To investigate whether the transition of H3K18cr pattern is associated with gene expression during early embryogenesis, we first examined ZGA‐only genes and categorized them into eight temporal clusters (C1–C8) according to their expression pattern from the late two‐cell to blastocyst stages (Figure 3e). The promoter‐associated H3K18cr dynamics closely mirrored transcriptional changes of the corresponding genes, indicating that stage‐specific reconfiguration of H3K18cr accompanies precise transcriptional transitions throughout preimplantation development (Figure 3e). Furthermore, these stage‐specific gene clusters were significantly enriched for biological processes corresponding to their developmental timing. For example, clusters with blastocyst‐specific expression and high H3K18cr levels (C4 and C8) were enriched for functions related to embryonic and placental development, consistent with the formation of trophectoderm cells at the blastocyst stage. To further assess the role of H3K18 crotonylation in early embryonic transcription, we performed perturbation experiments using the H3K18R mutant and further analyzed data examining the effects of P300 knockdown and rescue by the P300‐I1394G mutant. Expression of the H3K18R mutant, which abolishes crotonylation at histone H3 lysine 18, markedly reduced global H3K18cr signals in two‐cell embryos (Figure S6a) and caused widespread transcriptional alterations (Figure S6b). Notably, many major ZGA genes were downregulated upon H3K18R expression (Figure S6c,d), with stronger repression observed for genes whose promoters exhibit high H3K18cr enrichment (Figure S6e). Because P300 has been reported to possess histone crotonyltransferase activity, we analyzed a previously published dataset [8] comparing P300 siRNA embryos with embryos rescued by expression of the P300‐I1394G mutant after P300 knockdown (P300‐I1394G + P300 siRNA). The P300‐I1394G mutant, which lacks histone acetyltransferase activity but retains crotonyltransferase function, partially restored gene expression and promoter‐associated H3K18cr signals (Figure S6f). Notably, 8‐cell ZGA genes with strong promoter H3K18cr enrichment showed reduced expression and H3K18cr signals upon P300 knockdown but were partially restored in P300‐I1394G + P300 siRNA embryos (Figure S6g). Consistently, 43.6% (331 of 759) genes downregulated upon P300 knockdown overlapped with 8‐cell ZGA genes (Figure S6h). Moreover, genes with strong promoter H3K18cr signals exhibited greater transcriptional recovery in the rescue condition (Figure S6i). Taken together, these results suggest that the dynamics of narrow H3K18cr patterns may contribute to ZGA gene expression during early embryonic development, highlighting that the transition from broad to narrow domains serves as a prerequisite for H3K18cr‐mediated regulation of embryonic genome activation.

**FIGURE 4:**
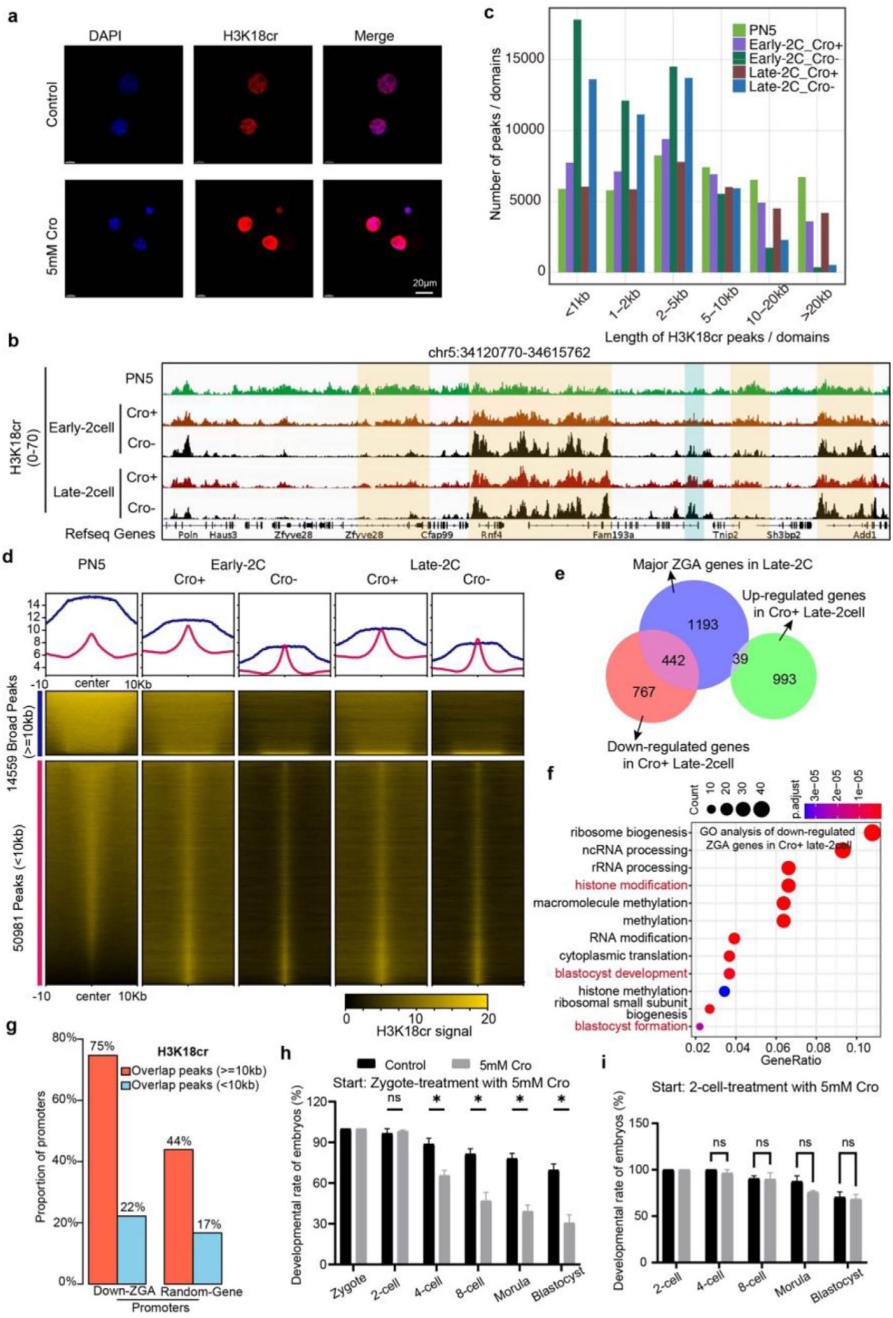
Transition of H3K18cr pattern contributes to major ZGA gene expression. (a) Immunostaining of H3K18cr for mouse two‐cell embryos. Control (Treatment with water in G1) and 5 mM Cro (Treatment with 5 mM crotonic acid in G1) groups. Scale bar: 20 µm. (b) Genome browser view of H3K18cr enrichment in mouse zygotes and crotonic acid‐treated two‐cell embryos. Yellow shading highlights broad H3K18cr regions in the mouse zygote. Blue shading highlights narrow H3K18cr regions in mouse two‐cell embryos with a relatively week signal in the zygote. Cro+ indicates embryos treated with crotonic acid, whereas Cro− indicates untreated embryos (control or normal embryos). (c) The number of H3K18cr peaks or domains within different ranges of lengths in mouse zygote and crotonic acid‐treated two‐cell embryos. (d) Heatmap comparing H3K18cr signals on two groups of peaks among early two‐cell, late two‐cell, and those embryos after crotonic acid treatment. The peaks were combined from PN5 zygote, normal early, and late two‐cell embryos. (e) Venn diagram showing the number of overlapping genes between genes upregulated or downregulated in late two‐cell embryos after crotonic acid treatment and major ZGA genes. (f) GO analysis of ZGA genes downregulated in late two‐cell embryos after crotonic acid treatment. (g) Bar chart displaying the percentage of promoters of downregulated ZGA genes overlapped by broad (≥10 kb) or narrow (<10 kb) H3K18cr peaks. (h, i) Statistical analysis of the effect of starting 5 mM crotonic acid treatment at the zygote (left) (h) or two‐cell (right) (i) stage on embryo development rate (n = 3 biological replicates) in mice. Statistical analysis was performed using multiple unpaired t‐tests. Developmental stages: two‐cell (p = 0.6676), four‐cell (p = 0.0059), eight‐cell (p = 0.0031), morula (p = 0.0006), blastocyst (p = 0.0019) for 5 mM Cro vs Control (h); four‐cell (p = 0.1773), eight‐cell (p = 0.8610), morula (p = 0.0339), blastocyst (p = 0.6574) for 5 mM Cro vs Control (i). The data were presented as Mean ± SEM of three independent biological replicates. ns (No significant difference), *p < 0.05.

To further verify that the transition of histone crotonylation from broad to narrow peak serves as a prerequisite for subsequent H3K18cr‐mediated regulation of later zygotic and embryonic genome activation, we treated mouse embryos with 5 mM crotonic acid starting from the zygote stage. Crotonic acid serves as a key precursor for crotonyl‐CoA synthesis, and its supplementation elevates intracellular crotonyl‐CoA levels, thereby promoting histone crotonylation and counteracting decrotonylation. We first evaluated the effects of different concentrations of crotonic acid on embryonic development. Embryos were treated with 0.05 mM, 0.5 mM, or 5 mM crotonic acid from the zygote stage, and developmental progression was monitored. While 0.05 mM and 0.5 mM crotonic acid had minimal effects on developmental rates, 5 mM crotonic acid markedly impaired embryonic development from the 4‐cell stage onward (Figure S7a). Based on these results, 5 mM crotonic acid was used in subsequent experiments to elevate histone crotonylation during early embryogenesis. As expected, immunofluorescence revealed a marked increase in H3K18cr in two‐cell embryos after crotonic acid treatment (Figure 4a). At the chromatin level, abundant broad H3K18cr peaks persisted in early and late two‐cell embryos treated with crotonic acid (Figure 4b,c). The aggregate signal of these peaks was most pronounced at the PN5 stage and gradually declined thereafter, indicating that crotonic acid delays but does not fully block the removal of broad H3K18cr domains during the two‐cell period. Concomitantly, narrow peaks that newly emerged at the two‐cell stage and exhibited weak signals within PN5 broad domains were still robustly established in crotonic acid‐treated embryos (Figure 4b,d). Collectively, these results indicate that the contraction or removal of PN5‐derived broad peaks and the de novo establishment of narrow peaks at new genomic loci are two independent processes. To assess the transcriptional impact of crotonic acid treatment, we profiled gene expression in early and late two‐cell embryos. Principal component analysis (PCA) showed that crotonic acid‐treated embryos and control early two‐cell embryos clustered closely together (Figure S7b), and only 8 minor‐ZGA genes were significantly downregulated at this stage (Figure S7c). In contrast, 442 major ZGA genes were markedly downregulated in late two‐cell embryos following crotonic acid exposure, with significant enrichment for pathways related to histone modification, blastocyst development, and formation (Figure 4e,f). Notably, 75% of these downregulated ZGA gene promoters were overlapped by broad H3K18cr domains, supporting a functional connection between impaired broad‐to‐narrow H3K18cr domain transition and compromised activation of major ZGA genes (Figure 4g). To further evaluate the developmental consequences of this transition, we treated embryos with crotonic acid beginning either at the PN5 stage, when broad H3K18cr domains predominate, or at the early two‐cell stage, when narrow peaks have already been established. Crotonic acid treatment initiated at PN5 significantly reduced the blastocyst formation rate, whereas treatment starting at the two‐cell stage had little effect on embryonic development (Figure 4h,i). These results indicate that the transition of broad H3K18cr domains during the PN5‐to‐early two‐cell window is essential for proper major ZGA activation and subsequent preimplantation development.

### 2.6 HDAC1‐Mediated Histone Deacetylase Activity Is Required for the Broad‐to‐Narrow Transition of Histone Crotonylation During Early Embryogenesis

**FIGURE 5:**
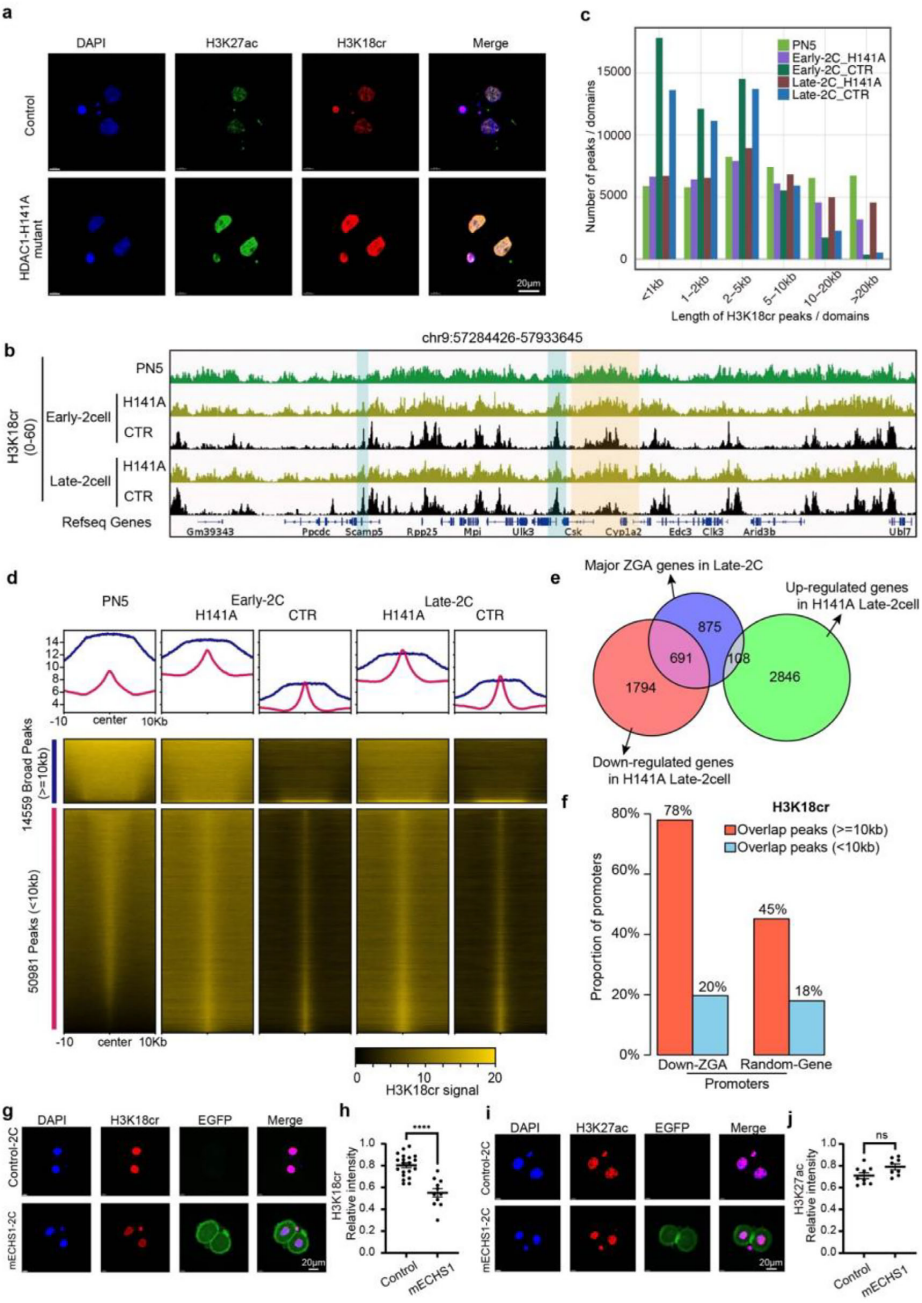
HDAC1‐mediated histone deacetylation activity is essential for the removal or transition of broad H3K18cr domains and ZGA in mouse. (a) Immunofluorescence staining of H3K27ac and H3K18cr in mouse two‐cell embryos. Control (water was injected) and HDAC1‐H141A (HDAC1‐H141A mutant mRNA was injected) groups. Scale bar: 20 µm. (b) Genome browser view of H3K18cr enrichment in mouse zygotes, HDAC1‐H141A mutant overexpressed two‐cell embryos, and control two‐cell embryos (CTR, normal embryos). Yellow shading highlights broad H3K18cr regions in the mouse zygote. Blue shading highlights narrow H3K18cr regions in mouse two‐cell embryos. (c) Bar plot showing the numbers of H3K18cr peaks or domains of different lengths in mouse zygotes, HDAC1‐H141A‐overexpressing two‐cell embryos, and control two‐cell embryos. (d) Heatmap comparing the H3K18cr signal on two groups of peaks among embryos at early two‐cell, late two‐cell stages, and after overexpression of the HDAC1‐H141A mutant. The peaks were combined from PN5 zygote, normal early, and late two‐cell embryos. (e) Venn diagram showing the overlap between genes upregulated or downregulated in late two‐cell embryos after HDAC1‐H141A overexpression and major ZGA genes. (f) Bar chart showing the percentage of promoters of downregulated ZGA genes (HDAC1‐H141A mutant) that were overlapped by broad (≥10 kb) or narrow (<10 kb) H3K18cr peaks. (g) Immunostaining of H3K18cr and EGFP for mouse two‐cell embryos. Control‐2C (water was injected) and mEchs1‐2C (*Echs1* mRNA with EGFP was injected) groups. Scale bar: 20 µm. (h) Dot plot comparing H3K18cr relative fluorescence intensity between control‐2C and *Echs1*‐2C groups (n = 20, n = 11). A *t*‐test was used. ^****^ *p* < 0.0001. (i) Immunostaining of H3K27ac and EGFP for mouse two‐cell embryos. Control‐2C (water was injected) and mECHS1‐2C (*Echs1* mRNA with EGFP was injected) groups. Scale bar: 20 µm. (j) Dot plot comparing H3K27ac relative fluorescence intensity between control‐2C and mECHS1‐2C groups (n = 9, n = 8). A *t*‐test was used.

The transition of histone crotonylation from broad to narrow peaks involves a pronounced decrotonylation process. Previous studies have shown that histone crotonylation and acetylation share common regulatory enzymes, including P300 and HDAC family members. Notably, histone acetylation also undergoes a broad‐to‐narrow peak transition from the zygote to the two‐cell stage [38]. These observations prompted us to test whether histone acetylation or its regulators might influence H3K18cr reprogramming. Among HDAC family members, HDAC1 emerged as the most plausible candidate. Biochemical study has identified HDAC1/2/3 as major histone decrotonylases in mammalian cells [13]. Analysis of published transcriptomic datasets [38] further revealed that *Hdac1* mRNA was markedly upregulated from the zygote to the two‐cell stage, coinciding with the global erasure of H3K18cr, whereas *Hdac2* expression gradually decreased and *Hdac3* remained at low levels (Figure S8a). In addition, previous functional study showed that *Hdac1* depletion causes pronounced defects or developmental delays during early embryogenesis, whereas knockdown of *Hdac2* or *Hdac3* results in minimal effects at this stage [39]. In addition, HDAC1 has been reported to regulate cell cycle progression and zygotic genome activation during preimplantation development, whereas HDAC2 primarily regulates chromosome segregation and oocyte development [40]. Together, these analyses and previous findings suggest that HDAC1 may act as a key candidate regulator of H3K18cr reprogramming during the zygote‐to‐two‐cell transition. To directly test this possibility, we constructed an HDAC1‐H141A mutant in which histidine 141 (H141) was substituted with alanine (A), a mutation reported to abolish its deacetylase activity through competitive inhibition without disrupting interactions with HDAC1‐associated proteins [41, 42]. Consistent with previous reports that broad histone acetylation domains are largely retained at the two‐cell stage following overexpression of this mutant [42], we also observed increased global histone acetylation levels (Figure 5a). Interestingly, H3K18cr levels were also significantly elevated in embryos overexpressing the HDAC1‐H141A mutant (Figure 5a). At the chromatin level, abundant broad H3K18cr peaks persisted in both early and late two‐cell embryos overexpressing the mutant (Figure 5b,c). The aggregate intensity of these broad peaks in early two‐cell embryos was comparable to that observed at the PN5 stage and gradually decreased in late two‐cell embryos (Figure 5d). Concomitantly, narrow peaks that newly emerged at the two‐cell stage and exhibited weak signals within PN5 broad domains were still robustly established in embryos overexpressing the HDAC1‐H141A mutant (Figure 5d), further confirming that the removal of PN5‐derived broad peaks and the de novo establishment of narrow peaks at new genomic loci are two independent processes. Together, these results demonstrate that the broad‐to‐narrow transition of H3K18cr depends on HDAC1‐mediated histone deacetylase activity.

Further transcriptome analysis revealed that the expression of a subset of 691 ZGA genes was downregulated in late two‐cell embryos overexpressing the HDAC1‐H141A mutant (Figure 5e). These downregulated ZGA genes were significantly enriched for pathways involved in histone modification, ribosome biogenesis, and other essential cellular processes (Figure S8b). Notably, 78% of these downregulated ZGA gene promoters overlapped with broad H3K18cr domains (Figure 5f; Table S3). Functional enrichment analysis of these genes revealed significant overrepresentation of processes related to transcriptional regulation, RNA processing, and early embryonic development, supporting a functional connection between impaired broad‐to‐narrow H3K18cr transition and compromised activation of major ZGA genes. This highlights a potential regulatory interplay between histone acetylation and crotonylation reprogramming that is essential for zygotic genome activation during early embryogenesis. To further determine whether histone crotonylation can in turn affect histone acetylation, we overexpressed ECHS1, an important metabolic hydratase enzyme that negatively regulates crotonyl‐CoA production and histone crotonylation (Figure S8c), by injecting ECHS1 mRNA into zygotes. As expected, overexpression of ECHS1 significantly reduced H3K18cr levels in the embryos (Figure 5g,h; Figure S8d), but did not alter histone acetylation (H3K27ac) levels (Figure 5i,j), which is consistent with its reported role in regulating H3K18cr in cardiac metabolism [17].

### 2.7 Histone Crotonylation Correlates with First Lineage Differentiation and Regulates the Blastocyst Formation

**FIGURE 6:**
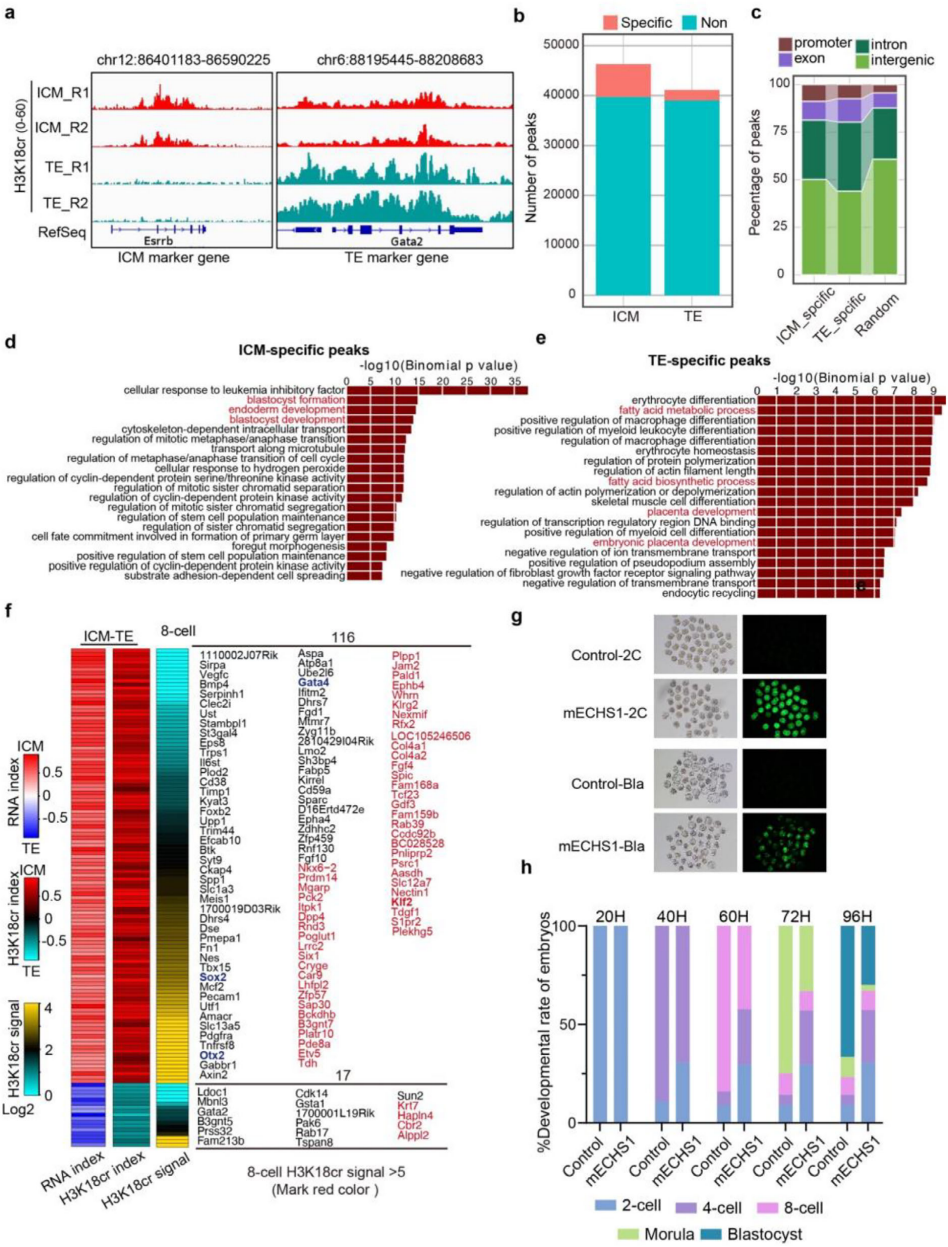
H3K18cr links to lineage differentiation and contributes to blastocyst formation. (a) Genome browser view of H3K18cr signal around the *Esrrb* gene (ICM marker/biased gene) and *Gata2* (TE marker/ biased gene) loci in ICM and TE samples. (b) Numbers of specific and non‐specific H3K18cr peaks in the ICM and TE. (c) Percentage of ICM‐ and TE‐specific H3K18cr peaks across different types of genomic elements. (d, e) GO analysis of genes associated with ICM‐specific (d) or TE‐specific (e) H3K18cr peaks. (f) Heatmap showing promoter‐associated ICM‐ and TE‐specific H3K18cr signals and gene expression levels, together with H3K18cr enrichment at the eight‐cell stage. The expression or H3K18cr index for ICM and TE was calculated as (ICM − TE) / (ICM + TE). (g) Representative images of two‐cell and blastocyst stages after injection of *Echs1* mRNA with EGFP. (h) The stacked bar chart shows the percentage of embryos in control‐2C and mEchs1‐2C groups that have developed to different stages (n = 3 independent replicates).

It is well‐known that expression of ZGA genes is critical for blastocyst development and lineage specification. Our abovementioned findings revealed an interplay between H3K18cr and ZGA, and stage‐specific H3K18cr‐associated genes were enriched for pathways related to blastocyst formation. To further investigate whether H3K18cr potentially regulates the first lineage differentiation of early embryos (inner cell mass, ICM, and trophectoderm, TE), we compared H3K18cr enrichment patterns between ICM and TE (Figure 6a,b), and found that the number and average length of ICM‐specific H3K18cr peaks were modestly greater than those of TE‐specific peaks (Figure 6b; Figure S9a). Moreover, loci encoding key lineage transcription factors, such as *Esrrb* for ICM identity and *Gata2* for TE, displayed corresponding ICM‐ and TE‐specific H3K18cr enrichment (Figure 6a). Most ICM‐ and TE‐specific H3K18cr peaks were located at distal regulatory regions or non‐promoter regions (Figure 6c; Figure S9b–e), which may associate with enhancer signals. Functional enrichment analysis further revealed that ICM‐specific H3K18cr peaks were associated with blastocyst and germ layer development (Figure 6d), whereas TE‐specific peaks were linked to fatty acid metabolism, biosynthesis, placental development, and ion transport (Figure 6e). To assess the transcriptional relevance of these modifications, we examined the expression of genes whose promoters showed ICM‐ or TE‐specific H3K18cr enrichment. Among 454 genes with ICM‐specific promoter H3K18cr marks, 116 (including *Sox2*, *Otx2*, *Gata4*, and *Klf2*) were highly expressed in the ICM (Figure 6f; Figure S9f). Similarly, among 111 genes with TE‐specific promoter H3K18cr, 17 (such as *Ldoc1*, *Gata2*, and *Cdk14*) showed elevated expression in the TE (Figure 6f; Figure S9g), while most other genes displayed both none or low expression, which may be due to the lack of necessary transcription factors. Interestingly, while many of these specifically marked genes were not yet transcriptionally activated, their H3K18cr enrichment may poise them for subsequent lineage‐specific expression in ICM‐ or TE‐derived lineages. Furthermore, a subset of ICM‐ and TE‐specific H3K18cr peaks already exhibited strong signals in earlier embryos (such as eight‐cell stage), suggesting that lineage specification might be epigenetically primed before morphological differentiation at the blastocyst stage. Genome browser views further showed that lineage‐specific H3K18cr enrichment at representative loci, including *Esrrb* and *Gata2*, frequently coincided with accessible chromatin regions (Figure S9h). Quantitative analysis revealed that 73% of ICM‐specific and 62% of TE‐specific H3K18cr‐marked promoters overlapped accessible chromatin regions (Figure S9i). In addition, functional perturbation analyses described above showed that expression of the P300‐I1394G mutant, a variant lacking histone acetyltransferase activity but retaining crotonyltransferase function, partially restored promoter‐associated H3K18cr levels together with the expression of corresponding genes following P300 knockdown (Figure S6f). Because gene activation generally requires both an accessible chromatin environment marked by active histone modifications and the timely binding of lineage‐specific transcription factors, these observations suggest that lineage‐specific H3K18cr enrichment may mark permissive regulatory regions in ICM and TE, supporting transcription either at the current developmental stage when appropriate transcription factors are present or at subsequent stages when additional lineage‐determining factors become available.

To further evaluate the role of histone crotonylation in blastocyst development, we overexpressed ECHS1, a key metabolic hydratase enzyme that negatively regulates crotonyl‐CoA production and histone crotonylation (Figure S8c), by injecting ECHS1 mRNA into zygotes. Fluorescence signals verify the elevated ECHS1 expression at the two‐cell and the blastocyst stages in the overexpressed ECHS1 groups (Figure 6g). As described above, overexpression of ECHS1 significantly reduced H3K18cr levels in the embryos without altering histone acetylation (H3K27ac) levels (Figure 5g–j), consistent with its reported role in regulating H3K18cr in cardiac metabolism [17]. Moreover, 30.49% and 26.83% of ECHS1‐overexpressing embryos were arrested at the two‐cell and four‐cell stages, respectively, and only 29.88% developed to the blastocyst stage, whereas 66.45% of control embryos successfully reached the blastocyst stage (Figure 6h). Notably, during the transition of H3K18cr from broad to narrow peaks, endogenous ECHS1 expression declined from the PN5 to the two‐cell stage, in line with its role as a negative regulator of histone crotonylation (Figure S9j). Together, these observations suggest that H3K18cr may contribute to lineage differentiation and blastocyst development during early embryogenesis.

In summary, H3K18cr undergoes extensive and stage‐specific reprogramming in mammalian gametes and early embryos. Its dynamic interaction with zygotic genome activation forms a key epigenetic mechanism that may orchestrate chromatin remodeling, drive transcriptional activation, and facilitate the successful progression from genome activation to the first lineage segregation during embryonic development.

## 3 Discussion

**FIGURE 7:**
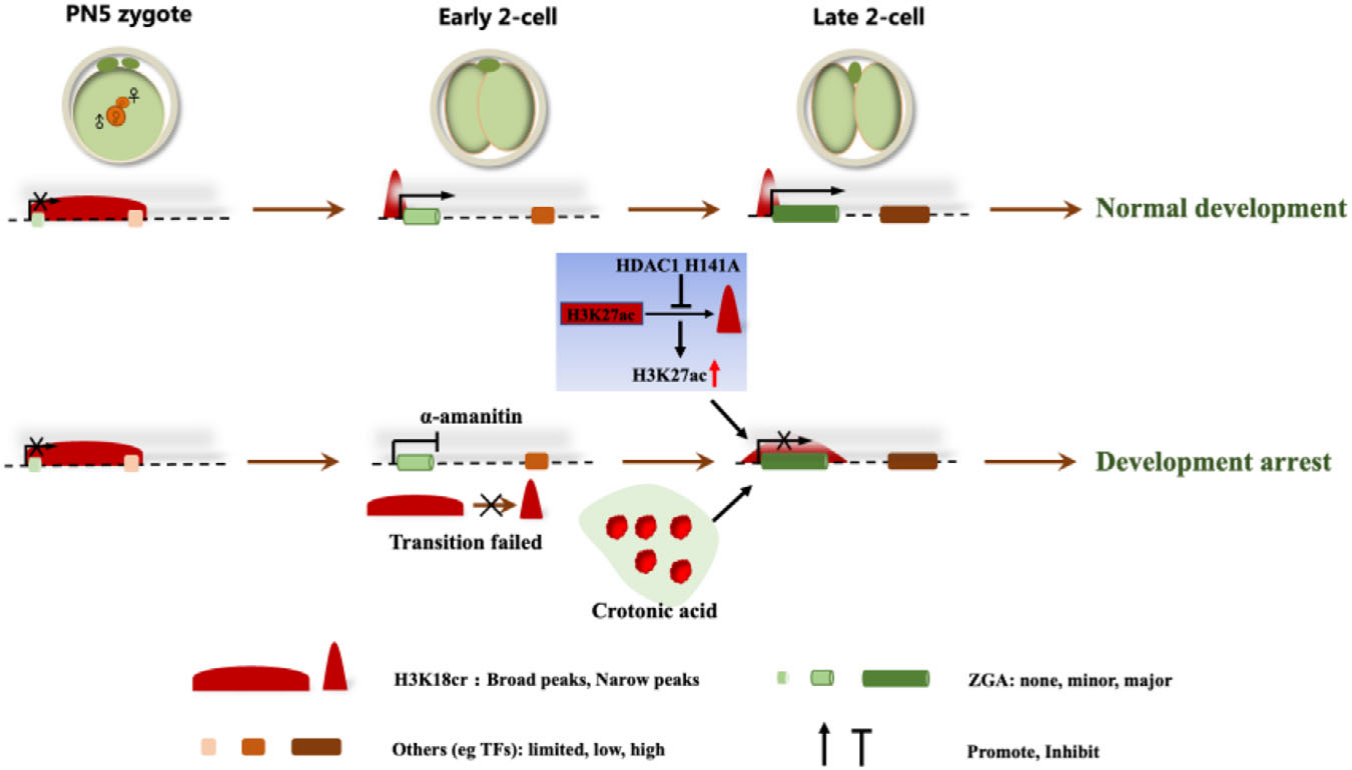
The working model illustrates the transition of H3K18cr domains and its regulations during preimplantation embryonic development. H3K18cr is crucial for proper embryonic development. Under normal conditions, H3K18cr undergoes a transition from broad peaks in the zygote to narrow peaks during the early two‐cell stage. This transition ensures the proper transcription of major ZGA genes, facilitating the embryo's progression to the blastocyst stage. Conversely, the broad‐to‐narrow transition of H3K18cr is transcription‐dependent during minor ZGA. Inhibition of zygotic transcription with α‐amanitin preserves broad PN5‐like domains and prevents their conversion into narrow peaks at the early two‐cell stage. Additionally, two orthogonal perturbations—elevating crotonic acid (a metabolic precursor of crotonyl‐CoA) and disabling HDAC1 deacetylase activity—both delay or reduce the loss of broad H3K18cr peaks, leading to attenuated major ZGA activation and impaired embryo development. This model underscores the link between H3K18cr turnover and transcriptional competence in preimplantation embryogenesis.

Our study delineates a dynamic and stage‐specific reprogramming of histone crotonylation during the earliest phases of mammalian development. H3K18cr is abundant in GV oocytes and sperm yet virtually absent in MII oocytes, and it is rapidly re‐established after fertilization. We further identify a distinctive chromatin transition, in which broad H3K18cr domains characteristic of zygotes are reshaped into canonical narrow peaks precisely coinciding with minor ZGA (Figure 7). Allele‐resolved analyses reveal stronger maternal H3K18cr in zygotes that progressively converges with the paternal genome, indicating distinct parental contributions that are subsequently harmonized as development proceeds. Mechanistically, the broad‐to‐narrow transition of H3K18cr is transcription‐dependent during minor ZGA. Inhibition of zygotic transcription with α‐amanitin preserved PN5‐like broad domains and prevented their conversion into narrow peaks (Figure 7), demonstrating that early transcriptional activity drives the remodeling of the crotonylation landscape. Two orthogonal perturbations, elevating crotonic acid (a metabolic precursor of crotonyl‐CoA) and disabling HDAC1 deacetylase activity, both delayed or reduced the loss of broad H3K18cr peaks and attenuated major ZGA activation (Figure 7), linking Kcr turnover to transcriptional competence. Notably, narrow peaks that formed de novo at the two‐cell stage persisted under these perturbations, indicating that the clearance of PN5‐derived broad domains and the establishment of new narrow peaks are partly independent processes governed by distinct regulatory mechanisms.

Promoter‐associated H3K18cr dynamics closely mirror temporal ZGA gene expression, with stage‐specific clusters showing coordinated promoter enrichment, particularly at CpG‐rich regions. Lineage‐biased H3K18cr enrichment is evident at ICM‐ and TE‐defining loci (such as *Esrrb* and *Gata2*), suggesting crotonylation primes chromatin for lineage specification before morphological segregation. Disruption of crotonyl‐CoA metabolism or HDAC1 complex function underscores the importance of balanced Kcr dynamics. Crotonic acid treatment delays the removal of broad H3K18cr domains, whereas ECHS1 overexpression reduces H3K18cr levels and impedes blastocyst development. These results suggest that timely decrotonylation, in concert with HDAC1‐dependent deacetylation, is crucial for chromatin resetting, proper genome activation, and subsequent lineage differentiation.

Although our analyses reveal an association between lineage‐specific H3K18cr enrichment and chromatin accessibility, the functional role of this modification remains to be fully clarified. Many ICM‐ or TE‐specific H3K18cr‐marked genes are not yet transcriptionally active at the examined stages, suggesting that H3K18cr enrichment alone may not be sufficient to initiate transcription. Instead, H3K18cr may mark permissive regulatory regions that become transcriptionally active upon recruitment of appropriate lineage‐specific transcription factors and chromatin regulators. In this context, H3K18cr may contribute to a primed chromatin state that facilitates subsequent lineage‐specific gene activation during later developmental stages.

Several limitations of this study should be acknowledged. First, although we employed an ultra‐low‐input chromatin profiling strategy, potential biases related to chromatin fragmentation, immunoprecipitation efficiency, and locus‐specific signal dropout cannot be fully excluded, and the measurements were derived from mixed‐cell populations. Single‐cell multi‐omic approaches will be essential to refine the temporal order of events and resolve cell‐type heterogeneity during early development. Second, α‐amanitin and metabolic interventions may have pleiotropic effects. Although swapping of the AGG sequence in HDAC1 with VRPP generates the HDAC1‐VRPP mutant can modulate Kcr levels in cell lines without affecting histone acetylation [13], our unpublished data show that this mutation does not alter H3K18cr levels in embryos, suggesting that embryonic H3K18cr regulation may involve additional or distinct mechanisms. Therefore, genetic perturbations that selectively alter histone crotonylation, rather than acetylation, in major regulatory enzymes such as P300, HDAC1/2, SIRT family, and YEATS‐domain proteins will be crucial for defining the specific contribution of Kcr to embryonic genome activation. Third, while mouse embryos provide mechanistic insight, comparative analyses using human embryos or embryo‐like models will be required to assess the conservation of these regulatory mechanisms, given species‐specific differences in ZGA timing. Future studies should identify the transcription factors and chromatin remodelers that recruit or repel Kcr during minor ZGA, define the reader proteins that interpret H3K18cr at promoters and enhancers, and map how metabolic flux into crotonyl‐CoA is integrated with developmental signaling. Locus‐specific manipulation of crotonylation, combined with live‐cell transcriptional reporters, may ultimately test whether the conversion from broad to narrow Kcr domains is sufficient to potentiate ZGA activity.

In sum, H3K18cr reprogramming emerges as a transcription‐dependent and enzyme‐tuned axis of early epigenetic control. Proper timing of broad‐domain resolution and focal peak establishment aligns with major ZGA, primes lineage‐specific regulatory elements, and supports successful progression to the blastocyst, thereby linking metabolic state, chromatin remodeling, and embryonic genome activation in a unified framework.

## 4 Experimental Section

### 4.1 Mouse Gametes and Early Embryo Collection

Oocytes and sperm were collected separately from C57BL/6J and DBA/2 mice. Sperm samples were incubated in IVF medium at 37°C in a 5% CO_2_ incubator for 30 min to eliminate somatic contaminants. The supernatant containing motile sperm was carefully transferred to a new centrifuge tube, and this incubation–transfer procedure was repeated twice. Sperm quality and concentration were assessed microscopically. Samples were centrifuged, the supernatant removed, and the pellet resuspended three times in 0.04% BSA solution. After the final wash, the supernatant was removed, and samples were snap‐frozen for storage. Fully grown germinal vesicle (GV)‐stage oocytes were obtained from 6‐week‐old C57BL/6J females 46–48 h after intraperitoneal injection of 10 IU pregnant mare serum gonadotropin (PMSG). Ovaries were transferred into COC medium, minced with a sterile blade, and GV oocytes were collected and transferred into G1 medium. Preimplantation embryos were collected from 6–7‐week‐old C57BL/6J females (SPF [Beijing] Biotechnology Co., Ltd.) mated with DBA males (SPF [Beijing] Biotechnology Co., Ltd.). To induce ovulation, females received 10 IU human chorionic gonadotropin (hCG) intraperitoneally 46–48 h after PMSG injection (San‐Sheng Pharmaceutical Co., Ltd.). Embryos at defined stages were recovered from the oviducts at specific time points after hCG administration: 13–15 h (MII oocyte), 26–27 h (PN5 zygote), 32–34 h (early two‐cell), and 46–47 h (late two‐cell). Four‐cell and eight‐cell embryos were obtained by culturing two‐cell embryos in G1 medium for 10 h or 16 h, respectively. Abnormal embryos were excluded. Cavitated E3.5 blastocysts were collected by flushing the uterine horns with COC medium. The zona pellucida of embryos selected by morphology or cell number was removed using G‐MOPS containing 0.8% HCl. Polar bodies were gently dissociated by pipetting with a narrow‐bore glass capillary. After washing in 0.04% BSA/PBS, embryos were used for ChIP‐seq analysis. At least two biological replicates were performed. Inner cell masses (ICMs) were isolated as previously described with slight modifications [32]. Briefly, E3.5 embryos were treated with G‐MOPS containing 0.8% HCl to remove the zona pellucida. After washing with 0.04% BSA/PBS, embryos were incubated in G1 medium containing rabbit anti‐mouse lymphocyte serum (Sigma, M5774; 1:5 dilution) for 30 min at 37°C. They were then washed and transferred into G1 medium containing guinea pig complement (Solarbio, S4990; 1:15 dilution) and incubated for 10 min at 37°C. Lysed trophectoderm cells were removed by gentle pipetting with a glass capillary. The remaining ICM clumps were washed in 0.04% BSA/PBS and snap‐frozen for downstream analysis. For trophectoderm (TE) collection, ICM and TE were separated by micromanipulation. TE clumps were washed in 0.04% BSA/PBS and snap‐frozen for subsequent assays.

### 4.2 In Vitro Fertilization (IVF)

For the functional validation experiments involving crotonic acid, HDAC1‐H141A, and H3K18R ICR mice (SPF [Beijing] Biotechnology Co., Ltd.) embryos derived from in vitro fertilization were used (Table S1). The cauda epididymis was dissected with scissors, and spermatozoa were squeezed out. After capacitation by incubation in IVF medium at 37°C in a 5% CO_2_ incubator for 30 min, the sperm were added into droplets containing MII oocytes. After 5 h of co‐incubation, the zygotes were transferred into G1 medium. All embryos were collected using a sterile pipette under a dissecting microscope to ensure precise timing and minimize contamination.

### 4.3 α‐Amanitin and Crotonic Acid Treatment of Mouse Embryos

Zygotes were collected 19 h after hCG injection and cultured in G‐1 PLUS medium (Vitrolife) supplemented with either sterile H_2_O (control) or 100 µM α‐amanitin (Sigma, A2263). For α‐amanitin treatment, embryos were cultured for 16 h at 37°C in a 5% CO_2_ incubator until reaching the early two‐cell stage (approximately 35 h after hCG). The treated embryos were harvested at the early two‐cell stage and used for H3K18cr ChIP‐seq analysis. For crotonic acid treatment, mouse zygotes (19 h after hCG) were cultured in G‐1 PLUS medium containing either sterile H_2_O (control) or 5 mM crotonic acid (Sigma, 130488) for 16 h or 28 h, corresponding to the early two‐cell and late two‐cell stages, respectively. Embryos were collected at the indicated stages and processed for H3K18cr ChIP‐seq or immunostaining assays.

### 4.4 In Vitro Transcription and Microinjection of *Hdac1‐H141A*, *Echs1*, and *H3K18R* mRNA

T7 promoter sequences were introduced into the *Echs1* and *Hdac1* coding regions by PCR amplification using the expression vectors pCMV‐Echs1 (mouse)‐Neo (P65798, Miaolingbio Inc.), pCMV‐HDAC1 (mouse)‐3×FLAG‐Neo (P43340, Miaolingbio Inc.), pDC316‐H3f3a (mouse)‐3×FLAG (P48001, Miaolingbio Inc.) and pLV3‐CMV‐H3f3b (mouse)‐3×FLAG‐CopGFP‐Puro (P56081, Miaolingbio Inc.), together with primers T7‐Echs1, T7‐Hdac1, T7‐H3f3a and T7‐H3f3b, respectively. The *Hdac1‐H141A* and *H3K18R* constructs were generated using the Mut Express II Fast Mutagenesis Kit V2 (Vazyme, C214) following the manufacturer's protocol. All constructs were verified by Sanger sequencing (Sangon Biotech, Shanghai, China) prior to use. For mRNA synthesis, plasmid templates were amplified using Phanta Flash Super‐Fidelity DNA Polymerase (Vazyme, P521‐d2) and purified with the FastPure Gel DNA Extraction Mini Kit (Vazyme, DC301). The linearized DNA templates were transcribed in vitro using the mMESSAGE mMACHINE T7 Ultra Kit (Invitrogen, AM1345), purified with VAHTS RNA Clean Beads (Vazyme, N412‐01), and dissolved in nuclease‐free water. The resulting mRNAs were aliquoted and stored at −80°C until use. Microinjection was performed 19–22 h after hCG administration. Approximately 10 pL of mRNA was injected into the cytoplasm of zygotes using an Eppendorf PiezoXpert micromanipulator coupled with a CellTram Vario microinjector. *Hdac1‐H141A* mutant mRNA (500 ng/µL) or *Echs1* mRNA (1 µg/µL) or *H3K18R* mutant mRNA (200 ng/µL) was loaded into microinjection needles, and injection pressure was carefully adjusted to ensure consistent delivery. Injected embryos were cultured in G‐1 PLUS medium (Vitrolife) under humidified conditions at 37°C with 6% CO_2_ in air. Embryos injected with nuclease‐free water served as controls.

### 4.5 Immunostaining

After removal of the zona pellucida, mouse oocytes and embryos were washed three times in PBS and fixed in 4% paraformaldehyde (PFA) for 30 min at room temperature. Following three 5‐min washes in PBS, samples were permeabilized in PBS containing 0.3% Triton X‐100 for 60 min. Embryos were then blocked in PBS supplemented with 1% BSA, 0.1% Tween‐20, and 0.01% Triton X‐100 for 30 min at room temperature. Primary antibody incubation was performed overnight (approximately 12 h) at 4°C with anti‐H3K18cr or anti‐H3K27ac antibodies (1:200 dilution). After three washes in PBS containing 0.1% Tween‐20 and 0.01% Triton X‐100, embryos were incubated with Alexa Fluor 594‐conjugated goat anti‐rabbit IgG secondary antibody and DAPI for 30 min at room temperature. Samples were washed three additional times in PBS containing 0.1% Tween‐20 and 0.01% Triton X‐100, then mounted on glass slides using VECTASHIELD Antifade Mounting Medium with DAPI (Vector Laboratories). Confocal images were captured using a Zeiss LSM 710 microscope equipped with a 40× oil‐immersion objective. All staining experiments were performed independently at least twice. Image processing and fluorescence quantification were conducted using ImageJ software.

### 4.6 ULI‐NChIP‐seq Library Preparation for H3K18cr

Ultra‐low‐input native chromatin immunoprecipitation followed by sequencing (ULI‐NChIP‐seq) was performed as previously described with minor modifications [34, 35]. ULI‐NChIP‐seq was performed as previously described with minor modifications. Briefly, mouse embryos from defined developmental stages were collected in low‐binding PCR tubes and stored at −80°C until use. Ten µL Dynabeads Protein G (Life Technologies, 10003D) were washed twice with ice‐cold RIPA buffer (10 mM Tris‐HCl pH 7.5, 140 mM NaCl, 1 mM EDTA, 0.5 mM EGTA, 1% Triton X‐100, 0.1% SDS, 0.1% sodium deoxycholate, 1 mM PMSF, 1× protease inhibitor cocktail, 20 mM Na‐butyrate) and incubated with 1 µL anti‐H3K18cr antibody (PTM Biolabs, PTM‐540) for 3 h at 4°C. The antibody‐coated beads were washed twice and resuspended in 150 µL RIPA buffer for subsequent use. Embryos were lysed in 30 µL cold lysis buffer (50 mM Tris‐HCl pH 7.4, 10 mM NaCl, 3 mM MgCl_2_, 0.5% Triton X‐100, 0.1% sodium deoxycholate, 1 mM PMSF, 1× protease inhibitors, 20 mM Na‐butyrate) for 30 min on ice and digested with 20 µL MNase mix at 21°C for 10 min. The reaction was stopped by adding EDTA and detergents, followed by rotation in 140 mM ChIP RIPA buffer for 1 h at 4°C. The chromatin was incubated with antibody‐coupled beads for over 6 h at 4°C, then washed twice with RIPA buffer containing 300 mM and 500 mM NaCl and once with TE buffer (10 mM Tris‐HCl pH 8.0, 1 mM EDTA). Chromatin DNA was eluted with 100 µL ChIP elution buffer (10 mM Tris‐HCl pH 8.0, 5 mM EDTA, 300 mM NaCl, 0.5% SDS) containing 5 µL proteinase K (20 mg/mL) and 60 ng carrier RNA (TIANGEN) at 55°C for 2 h and 65°C for 4 h. DNA was purified using 1.8× SPRIselect beads (Beckman Coulter) and eluted in 50 µL TE buffer. Libraries were constructed using the NEBNext Ultra II DNA Library Prep Kit (NEB, E7645S), size‐selected for 250–600 bp fragments, and sequenced as 150 bp paired‐end reads on Illumina NovaSeq 6000 or X Plus platforms. Two biological replicates were performed for each developmental stage (Table S1).

### 4.7 RNA‐seq Library Preparation and Sequencing

The RNA‐seq libraries were generated from early mouse embryos using Smart‐seq2 with minor modifications [43, 44]. Mouse early two‐cell and late two‐cell embryos (10 embryos per sample; *n* = 3 biological replicates per group) were collected at 20 h and 32 h post‐fertilization, respectively. Total RNA was extracted in 2 µL of lysis buffer prepared by mixing 1 µL RNase inhibitor with 19 µL of 0.2% Triton X‐100. Reverse transcription was performed using the SuperScript IV Reverse Transcriptase kit (Thermo Fisher, 18090050) according to the manufacturer's protocol. The reaction mixture was incubated at 50°C–55°C for 10 min, followed by enzyme inactivation at 85°C for 10 min. For cDNA pre‐amplification, 12.5 µL KAPA HiFi HotStart ReadyMix (KAPA Biosystems), 0.25 µL ISPCR primer, and 2.25 µL RNase‐free water were added to each reaction. PCR amplification was performed with the following program: initial denaturation at 98°C for 3 min; 12 cycles of 98°C for 20 s, 67°C for 15 s, and 72°C for 6 min; and a final extension at 72°C for 5 min. Amplified cDNA libraries were pooled and purified using AMPure XP beads (Beckman Coulter) according to the manufacturer's instructions. Sequencing libraries were prepared with the TruePrep DNA Library Prep Kit (Vazyme, UTD522). Final libraries were sequenced on an Illumina NovaSeq X Plus platform with 150 bp paired‐end reads (PE150). Three biological replicates were performed for each developmental stage (Table S1).

### 4.8 ULI‐NChIP‐seq Data Analysis

Raw ULI‐NChIP‐seq reads were trimmed to 100 bp, and low‐quality sequences were filtered using Trimmomatic (v0.32) [45]. Clean paired‐end reads were aligned to the Mus musculus reference genome (mm10) using Bowtie2 (v2.3.4.2) [46]. Reads with mapping quality scores below 10 were discarded, and PCR duplicates were removed using Picard (v1.119). The reads for two biological replicates were merged to call peaks using MACS2 v2.1.4 [47]. Given the globally distinct enrichment landscapes across developmental and treatment conditions, MACS2 peak calling was performed using customized parameters for different sample type [28, 38, 48]. Specifically, for GV oocytes, PN5 zygotes, α‐amanitin–treated or crotonic acid–treated embryos, and HDAC1 mutant (H141A)–overexpressing embryos, peaks were identified with the parameters “–broad –SPMR –nomodel ‐p 0.001 –broad‐cutoff 0.01 –max‐gap 500”, and the remained peaks or domains within 5 kb were further merged as described [28, 48]. For sperm, MII oocyte, and early embryos ranging from the early two‐cell stage to the blastocyst, peaks were identified using the parameters “–broad –SPMR –nomodel”, and adjacent peaks with a summed per‐base RPKM greater than 2000 and located within 500 bp of each other were further merged. BigWig tracks of H3K18cr signals for visualization in Integrative Genomics Viewer (IGV) were generated using the bamCoverage function in the deepTools2 suite [49] with the parameters “–normalizeUsing RPKM –binSize 50”. RPKM values for the unfiltered peaks described above were calculated using bwtool, based on the BigWig files.

Because the broadly distributed H3K18cr signal tends to produce lower RPKM values in mouse GV oocytes, zygotes, and early/late two‐cell embryos under certain treatments than in embryos at later stages, we further normalized the H3K18cr signal between typical peaks and broad domains using a strategy similar to that described previously [28, 38, 48]. We assumed that genomic regions with the highest H3K18cr enrichment (e.g., promoters) represent fully modified loci and display comparable signal intensities across samples. For each sample, RPKM values of H3K18cr signals at promoters were calculated and ranked in descending order. The top 3,000 promoters were selected, and their median RPKM value (Median_(__s__i__)_) was defined as a sample‐specific scaling index. The median value of blastocyst samples (Median_(__Bla__)_) was used as a fixed reference (scaling factor = 1), and the scale factor for each other sample was calculated as Scale_(__s__x__)_ = Median_(__Bla__)_ / Median_(__s__i__)_. Unless otherwise specified, all H3K18cr signal intensities used for comparison and visualization were multiplied by the scale factor. Because the H3K18cr signal in MII oocytes was extremely weak, this normalization strategy was not applied to those samples.

Pearson correlation coefficients among biological replicates were computed using the multiBigwigSummary and plotCorrelation modules in deepTools [49]. Heatmaps and profile plots depicting H3K18cr signal distributions across genomic features (including peaks and promoters) were generated using the computeMatrix, plotHeatmap, and plotProfile modules of deepTools. The peaks with H3K18cr signal in ICM that are threefold greater than those in TE and with H3K18cr signal intensity ≥ 8 were regarded as ICM‐specific peaks, while the peaks with H3K18cr signal in TE that are threefold greater than those in ICM and with H3K18cr signal intensity ≥ 8 were regarded as TE‐specific peaks.

For promoters overlapping with ICM‐specific peaks, those with H3K18cr signal intensity in ICM that was twofold greater than in TE and with H3K18cr signal intensity ≥ 4 were defined as promoters with ICM‐specific H3K18cr modification. For promoters overlapping with TE‐specific peaks, those with H3K18cr signal intensity in TE that was twofold greater than in ICM and with H3K18cr signal intensity ≥ 4 were defined as promoters with TE‐specific H3K18cr modification.

### 4.9 Analysis of the Histone Methylation, DNA Methylation, CUT‐TAG, and ATAC‐seq in Mouse Early Embryos

The STAR ChIP–seq data of H3K4me3 and H3K27me3 for gametes and early embryos were obtained from two previous publications [30, 31] (GSE71434, GSE76687). To enable better comparison with H3K18cr, the general processing pipeline was performed in a manner similar to that used for H3K18cr. For H3K4me3, peaks in GV oocytes, MII oocytes, zygotes, and early 2‐cell embryos were called by MACS2 with the parameters “–broad –SPMR –nomodel –nolambda –max‐gap 500”, and adjacent peaks or domains located within 5 kb were further merged to define the final peaks or domains. H3K4me3 Peaks in sperm and other embryos marked majorly with narrow patter were called with the parameters “–broad –SPMR –nomodel –nolambda”, and adjacent peaks with a summed per‐base RPKM greater than 2500 (embryos) or 1500 (sperm) and located within 500 (embryos) or 200 (sperm) bp of each other were further merged. Considering that both broad and narrow patterns were present, H3K4me3 signals were normalized using a strategy similar to that described above for H3K18cr. For H3K27me3, peaks at all stages predominantly exhibited a broad pattern and were therefore called using MACS2 with the parameters “–broad –SPMR –nomodel –nolambda –max‐gap 1000”. Adjacent peaks or domains within 6 kb were further merged, and merged regions with a summed per‐base RPKM greater than 7000 and a median per‐base RPKM greater than 0.5 were defined as the final peaks or domains. For DNA methylation analysis, all processed methylation data were obtained from a previous study [23] (GSE56697). DNA methylation levels across genomic regions were calculated as described in our previous study [35]. The processed bigWig and peak files from the single‐cell NanoATAC‐seq2 dataset [50] were downloaded from Zenodo (record 14030067) and directly used for visualization and other analysis. The CUT&Tag data for H3K18cr were obtained from a previous study [8] (GSE241196). Upstream processing was performed similarly to the ULI‐NChIP‐seq described above. Promoter signal intensities were calculated using deepTools [49].

### 4.10 Allelic H3K18cr Patterns in Mouse Early Embryos

A subset of mouse embryos was obtained from crosses between female C57BL/6J mice and male DBA/2 mice. To achieve an allele‐specific H3K18cr signal, we utilized SNPs that differ between the two strains to identify the reads from the maternal or paternal genome. SNPsplit was used to distinguish paternal and maternal reads [51]. Paternal and maternal BigWig tracks of H3K18cr signals for visualization in IGV were generated using the bamCoverage function in the deepTools2 suite with the parameters “–normalizeUsing RPKM –binSize 50”. A similar scale factor‐based normalization strategy was applied to further normalize the parental genome signals. In addition, the ratio of total mapped reads between paternal and maternal read numbers was incorporated into the calculation of the final scale factors for parental genomes. The H3K18cr peaks supported by more than 50 allelic reads were used to define parental‐specific peaks. Peaks with a ratio of (Mat − Pat) / (Mat + Pat) ≥ 0.4 were regarded as maternal‐specific, whereas those with a ratio ≤ −0.4 were regarded as paternal‐specific.

### 4.11 RNA‐seq Data Analysis

The RNA‐seq data for MII oocyte, zygote, early two‐cell, late two‐cell, four‐cell, eight‐cell, and blastocyst were obtained from a previous publication [43] (GSE66390). The RNA‐seq data for TE and ICM were from GSE97778 [52]. The RNA‐seq data for late two‐cell embryos overexpressing HDAC1 (H141A) and corresponding controls were obtained from GSE182553 [42]. The RNA‐seq data for control embryos, P300 siRNA embryos, and embryos expressing the P300‐I1394G mutant following P300 siRNA treatment (P300‐I1394G + P300 siRNA) were obtained from a previous publication [8] (GSE241196). RNA‐seq libraries of early and late two‐cell embryos treated with crotonic acid or microinjected with mRNA encoding H3K18R or wild‐type H3 were generated in this study using the Smart‐seq2 protocol. Sequencing reads were trimmed using Trimmomatic (v0.32) to remove adapter sequences and low‐quality bases. Clean reads were aligned to the Mus musculus reference genome (mm10) using HISAT2 (v2.1.0) [53] and to the transcriptome using Salmon (v0.8.2) [54]. Gene expression levels were quantified as FPKM values using Cufflinks (v2.2.1) [55]. Differentially expressed genes (DEGs) were identified with DESeq2 (v1.18.0) [56] based on the raw read counts produced by Salmon. Genes with an adjusted *p* value < 0.05 and a fold change ≥ 2 were defined as DEGs. Furthermore, DEGs were refined by retaining only those genes with FPKM ≥ 1, indicating relatively high expression, and, unless otherwise specified, at least a twofold difference in FPKM values between the compared groups.

### 4.12 Mouse Minor ZGA and Major ZGA

Minor ZGA genes at the early two‐cell were defined as those with FPKM > 3 in early two‐cell embryos, FPKM < 1 in zygotes, and showing more than a threefold increase in expression in early two‐cell embryos compared to zygotes. Major ZGA genes at late two‐cell were defined as those with FPKM > 3 in late two‐cell embryos, FPKM < 3 in zygotes, and showing more than a threefold increase in expression in late two‐cell embryos compared to zygotes. ZGA‐only genes from the two‐cell to blastocyst stages were defined as those with FPKM > 3 at any stage between the two‐cell and blastocyst stages and FPKM < 1 in zygotes. 8‐cell ZGA genes were defined as genes with FPKM > 3 in 8‐cell embryos, FPKM < 3 in zygotes, and a greater than threefold increase in expression in 8‐cell embryos relative to zygotes.

### 4.13 Cluster Analysis

PCA analysis of all samples based on their RNA expression patterns was performed using the “prcomp” function in R (v4.2.3). K‐mean analysis for ZGA‐only genes was performed using the “Cluster 3.0” software.

### 4.14 GO and Transcription Factor Motif Enrichment Analyses

Gene Ontology and functional enrichment analyses of H3K18cr peaks were performed using the GREAT [57] (Genomic Regions Enrichment of Annotations Tool) program to associate genomic regions with putative target genes. For ZGA‐only genes associated with promoter H3K18cr, Metascape [58] was used for GO analysis. For downregulated ZGA genes in early and late two‐cell embryos treated with crotonic acid or in late two‐cell embryos overexpressing HDAC1 (H141A), GO enrichment analysis was performed using clusterProfiler (v4.6.2) [59]. The findMotifsGenome.pl script in HOMER [60] (v5.1) was used to perform transcription factor motif enrichment analysis.

### 4.15 Genomic Annotation

The mm10 refGene files downloaded from UCSC Table Browser were used for genome annotations. The annotation files for genomics elements, including promoters (TSS±2 kb), exons, and introns, were downloaded from UCSC Table Browser. Unless otherwise stated, each ChIP‐seq peak or domain was assigned to a genomic category if at least 30% of its length overlapped with any genomic element in that category, or if at least 30% of the genomic element's length overlapped with the peak. The priority order for genomic annotation was promoter, TES, exon, intron, and intergenic region. The analysis was performed using BEDTools (v2.27.1) [61]. Genomic transposable or repetitive elements annotated by RepeatMasker were downloaded from the UCSC Table Browser.

### 4.16 Statistical Analysis

Statistical analyses and plots were implemented with R (v4.2.3) (http://www.r‐project.org). The statistical methods used for analysis were described in the figure legends. The Wilcoxon rank‐sum test was used to assess the significance of differences in RNA expression and peak length. The Wilcoxon signed‐rank test was performed using the ‘wilcox.test’ function (two‐sided). Student's t‐tests were applied to compare histone modification fluorescence intensity and embryo developmental outcomes. No statistical methods were used to predetermine sample size.

## Author Contributions

S.Y., K.W., and T.H. conceived the study. S.Y., Y.Y., and C.L. facilitated its designs. Y.Y., X.W., and K.Z. collected mouse samples and performed functional experiments. S.Y. and Y.Y. performed ULI‐NChIP‐seq library construction. Y.Y. performed RNA‐seq library construction. S.Y., Z.F., and Y.L. performed the bioinformatics analyses. K.W., S.Y., Y.Y., and T.H. interpreted the data. S.Y., Y.Y., X.W., and K.W. wrote the paper with the assistance of the other authors.

## Conflicts of Interest

The authors declare no conflicts of interest.

## Ethics Statement

All animal experiments were conducted following the guidelines of the Animal Care and Use Committee at Shandong University (ethical approval number: 2022–138).

## Supporting information




**Supporting File 1**: advs75143‐sup‐0001‐SuppMat.docx.

**Supporting File 2**: advs75143‐sup‐0002‐TableS1–S3.zip.

## Data Availability

ULI‐NChIP‐seq data and RNA‐seq data generated in this study have been deposited in the Genome Sequence Archive (GSA) with the accession number: CRA032027 in PRJCA049131. Processed H3K18cr ULI‐NChIP‐seq data from mouse gametes to blastocyst‐stage embryos, including normalized bigWig files and peak files, have been deposited in Zenodo (https://zenodo.org/records/18983574).
