# Supplementary material for: The Landscape and Regulation of Histone Crotonylation in Mammalian Gametes and Early Embryos
Source: Adv Sci (Weinh). 2026 Apr 3;13(36):e75143. doi: 10.1002/advs.75143 (PMC13317631; doi:10.1002/advs.75143)
Supplement: Supplementary file 1 — Supporting File 1: advs75143‐sup‐0001‐SuppMat.docx. [file ADVS-13-e75143-s002.docx]

**The landscape and regulation of histone crotonylation in mammalian gametes and early embryos**

Shenli Yuan^1,4^, Yelian Yan^2,4^, Yalin Liang^1^, Kanghua Zhong^1^, Zhican Fu^3^, Xiaobo Wang^1*^, Chao Liu^1*^, Tao Huang^2*^, Keliang Wu^2*^

^1^GMU-GIBH Joint School of Life Sciences, Guangzhou Women and Children's Medical Center, Guangzhou Medical University, Guangzhou, Guangdong, 511436, China

^2^State Key Laboratory of Reproductive Medicine and Offspring Health, Center for Reproductive Medicine, Institute of Women, Children and Reproductive Health, Shandong University, Jinan, Shandong, 250012, China

^3^Beijing Life Science Academy, Key Laboratory of Tobacco Biological Effects, Beijing, 102209, China.

^4^These authors contributed equally: Shenli Yuan, Yelian Yan

^*^Corresponding authors: Xiaobo Wang, Chao Liu, Tao Huang, and Keliang Wu

E-mail: wxiaobo113@163.com, liuchao@gwcmc.org, htao1568@126.com, [wukeliang_527@163.com](mailto:wukeliang_527@163.com)

This file includes:

Figure. S1 to S9

Additional Files:

Table S1: The sample information of mouse gametes and embryos used in ULI-NChIP-seq and Smart-seq2.

Table S2: The number of peaks with different length.

Table S3: GO analysis and ZGA genes with promoters covered by H3K18cr domains that are downregulated in HDAC1-H141A mutant embryos.

**Supplementary Figures**


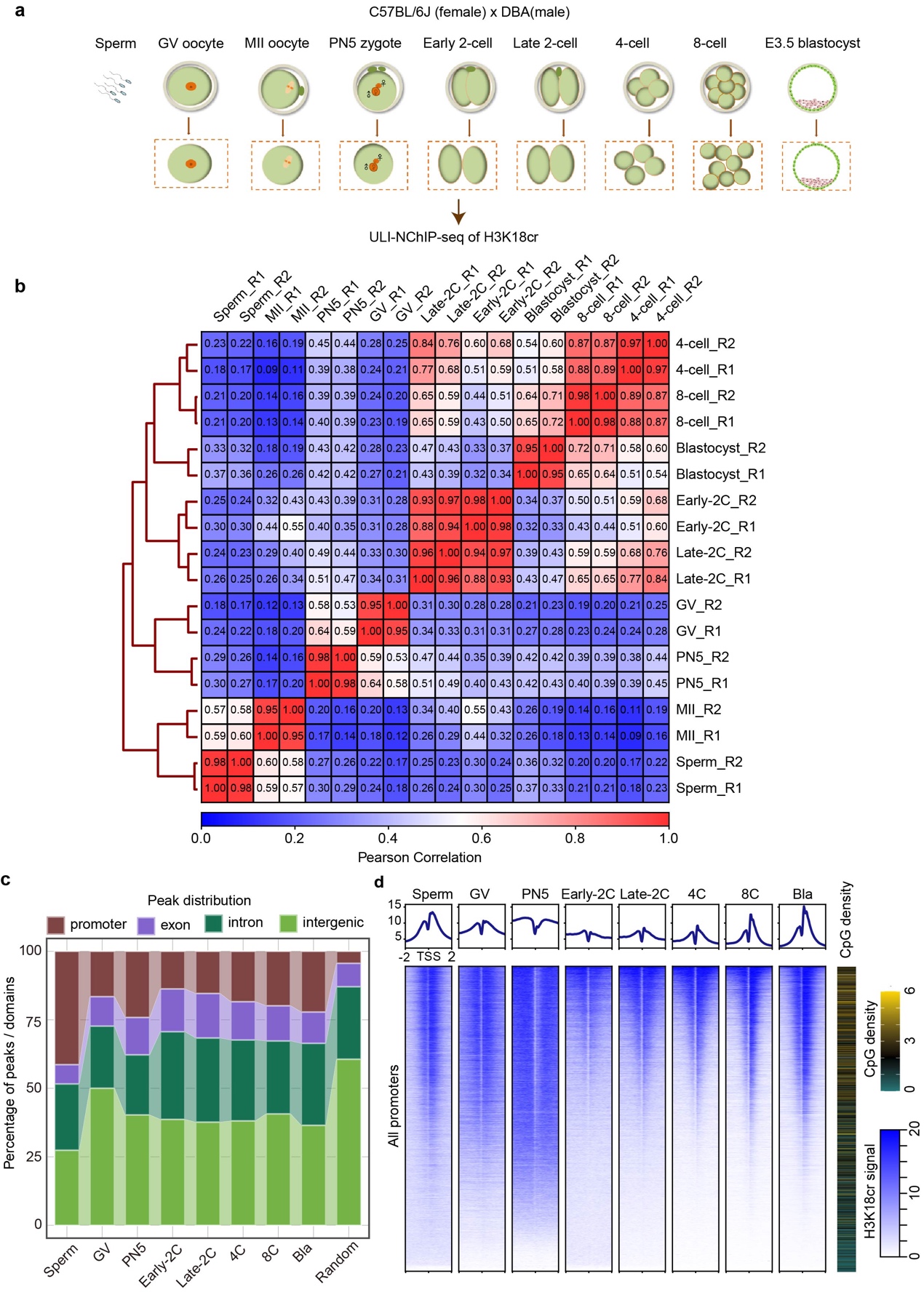


**Figure S1 |** **Correlation analysis and genomic element features of ULI-NChIP-seq data in mouse gametes and early** **embryos. a,** Schematic diagram of mouse gametes and early embryos collected for H3K18cr ULI-NChIP-seq. **b,** Heatmap showing correlations between the biological replicates. **c**, Percentage of H3K18cr peaks in different types of genomic elements at the indicated stages in mouse gametes and early embryos. **d**, Heat maps showing dynamics of H3K18cr signal at the gene promoters in mouse gametes and early embryos. The CpG densities of promoters are shown in the right.


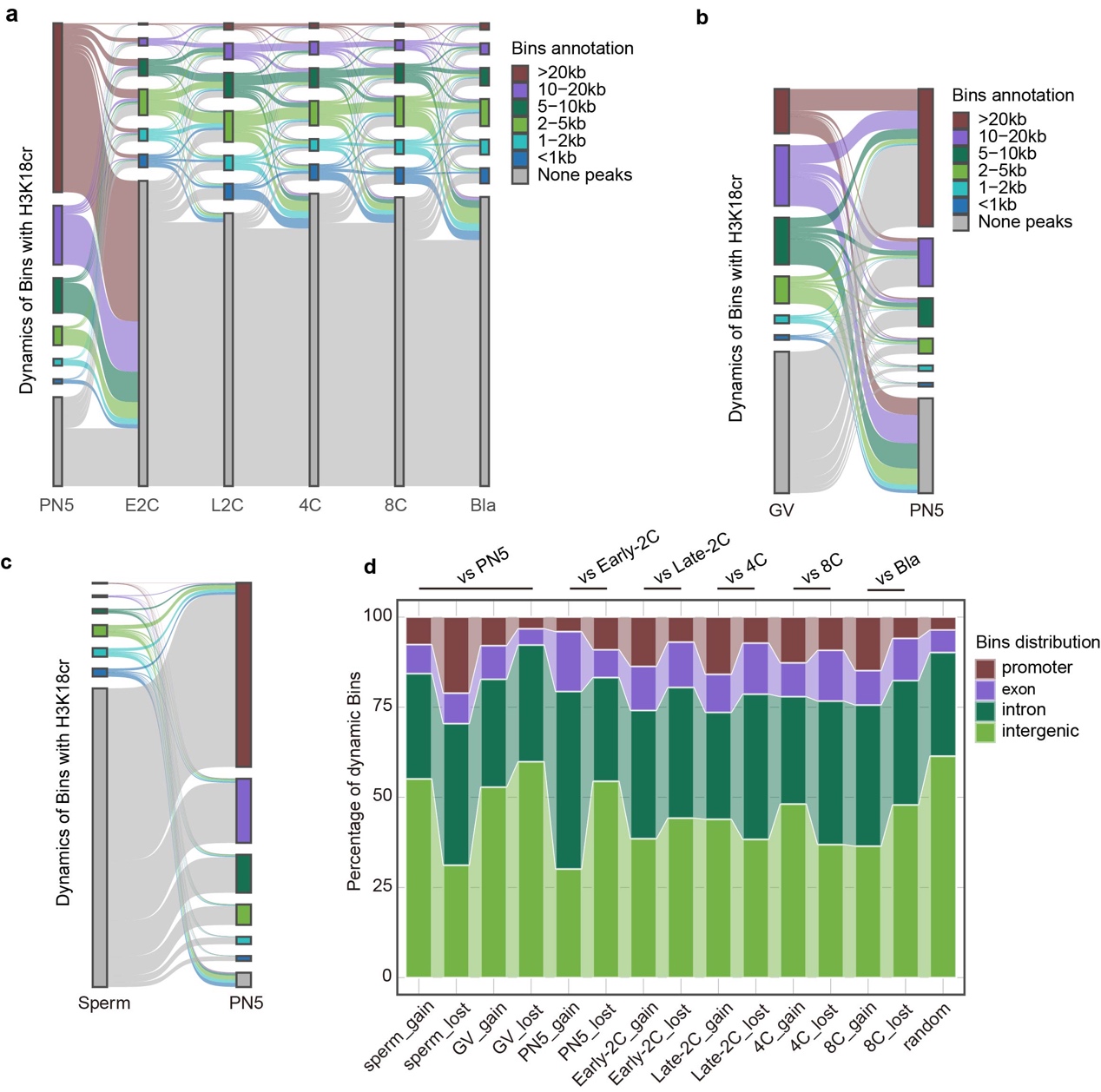


**Figure S2 |** **Characteristics of H3K18cr peaks in mouse gametes and early embryos. a,** Sankey diagram showing the dynamics of bins (1kb) with H3K18cr peaks with different ranges of peak lengths at each stage in mouse early embryos. E2C represents early 2-cell (two-cell) embryo. L2C represents late 2-cell (two-cell) embryo. **b,** Sankey diagram showing the dynamics of bins (1kb) with H3K18cr peaks with different ranges of peak lengths between GV oocyte and PN5 zygote in mouse. **c,** Sankey diagram showing the dynamics of bins (1kb) with H3K18cr peaks with different ranges of peak lengths between sperm and PN5 zygote in mouse. **d,** Bar plot comparing the distribution of genomic bins with dynamic H3K18cr changes across genomic elements. Bins were classified into Gain and Lost groups based on H3K18cr signal changes between two stages. For example, sperm_gain represents bins with enriched H3K18cr signal in sperm but not in PN5 zygote, while sperm_lost represents bins without enriched H3K18cr signal in sperm but with enrichment in PN5 zygote.


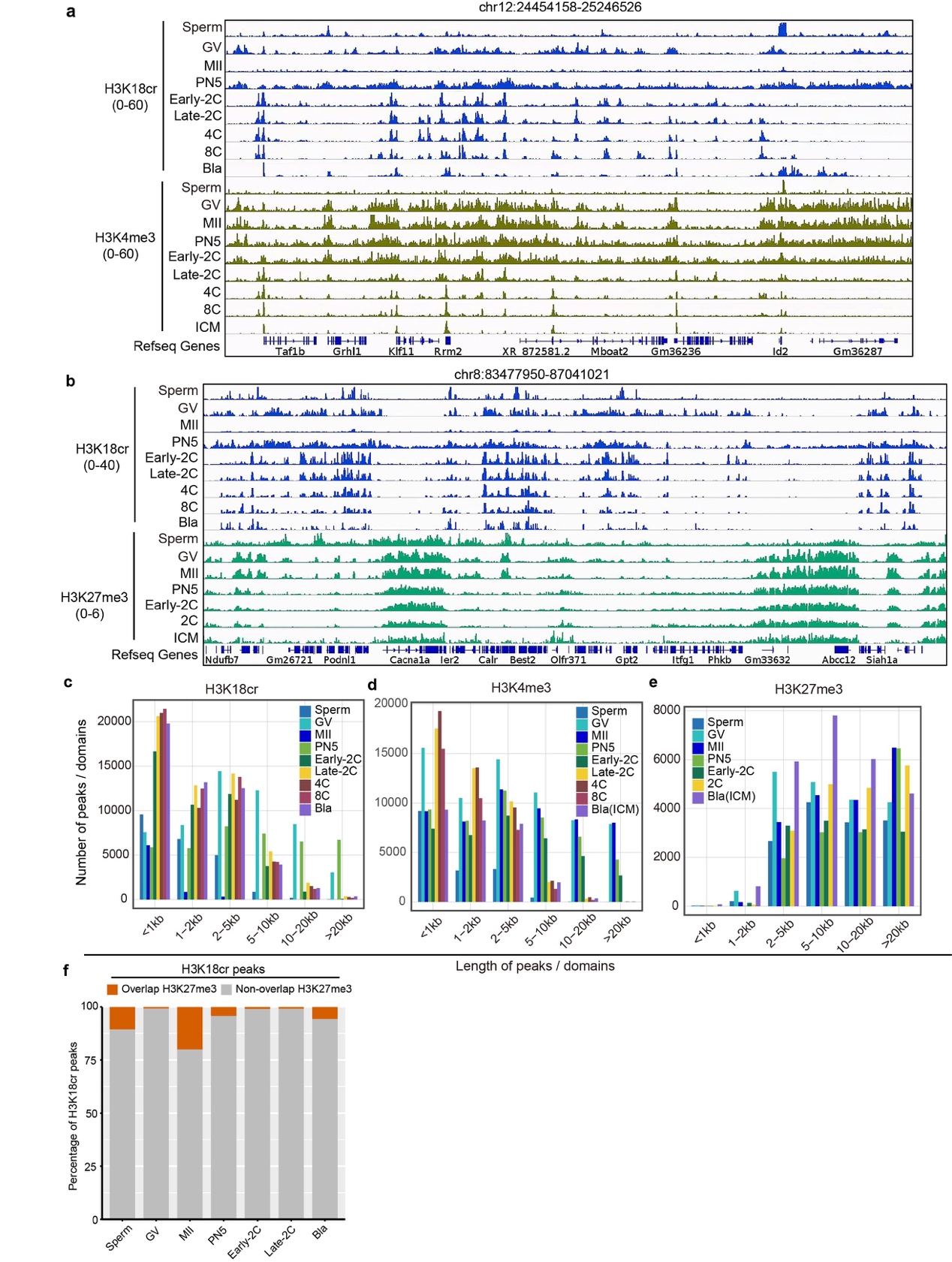


**Figure S3 | Comparison of H3K18cr signal with histone methylation in mouse gametes and early embryos. a-b,** Genome browser views of H3K18cr, H3K4me3 (a), and H3K27me3 (b) signals. **c-e,** The numbers of H3K18cr (c), H3K4me3(d), H3K27me3 (e) peaks or domains with different ranges of lengths in mouse gametes and early embryos. **f,** Percentage of H3K18cr peaks that overlap or do not overlap with H3K27me3 peaks at the indicated stages in mouse gametes and early embryos.


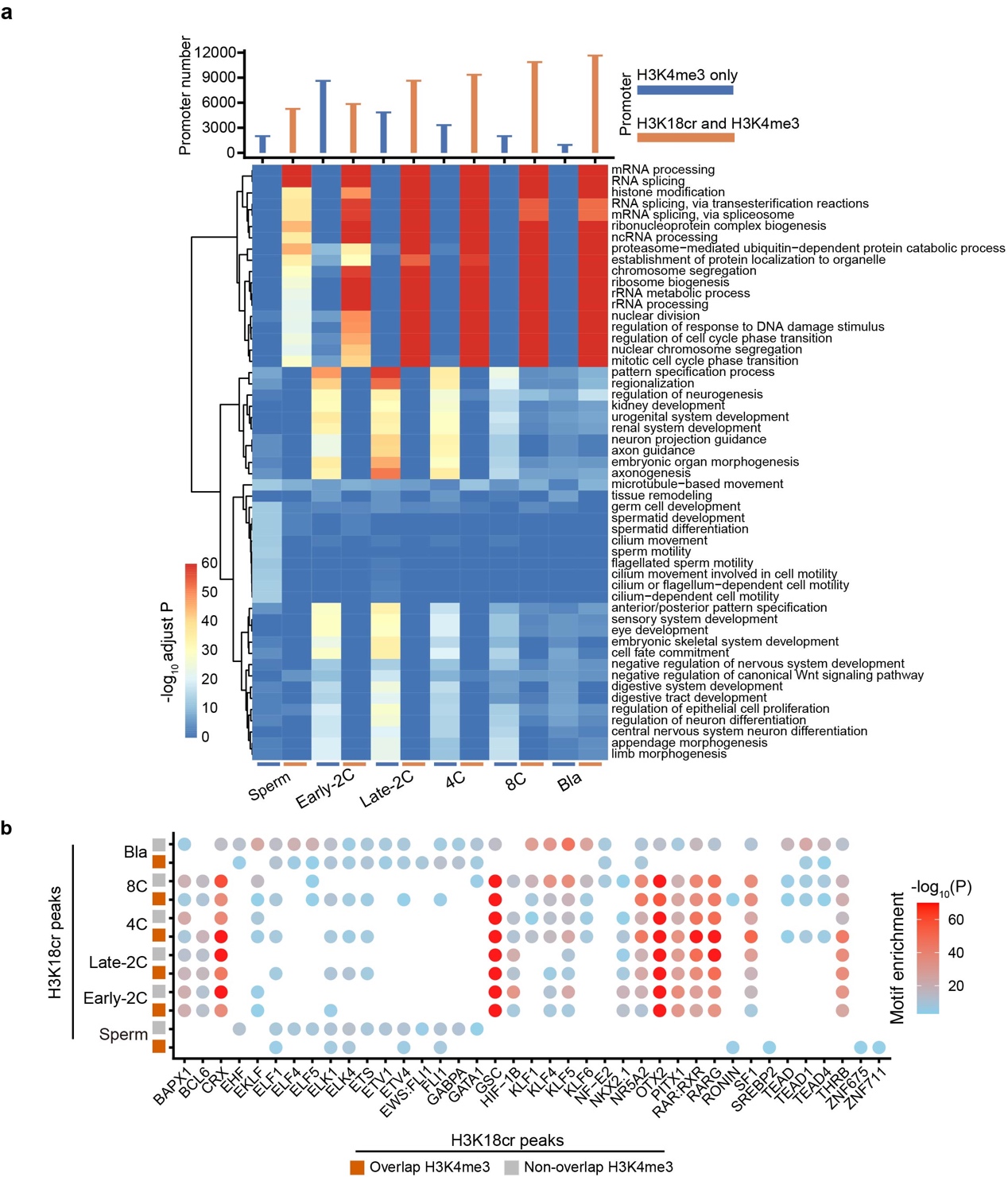


**Figure S4 | Functional annotation and transcription factor motif enrichment analysis of H3K18cr peaks that overlap or do not overlap with H3K4me3 peaks at the indicated stages in mouse gametes and early embryos. a,** GO analysis of genes associated with promoter peaks that either overlap between H3K18cr and H3K4me3 or are marked by H3K4me3 alone (only) at the indicated stages. The upper panel shows the number of promoters in each category, and the lower heatmap shows the enrichment significance of representative biological processes. **b,** Transcription factor motif enrichment analysis of H3K18cr peaks that overlap or do not overlap with H3K4me3 peaks across the indicated stages.


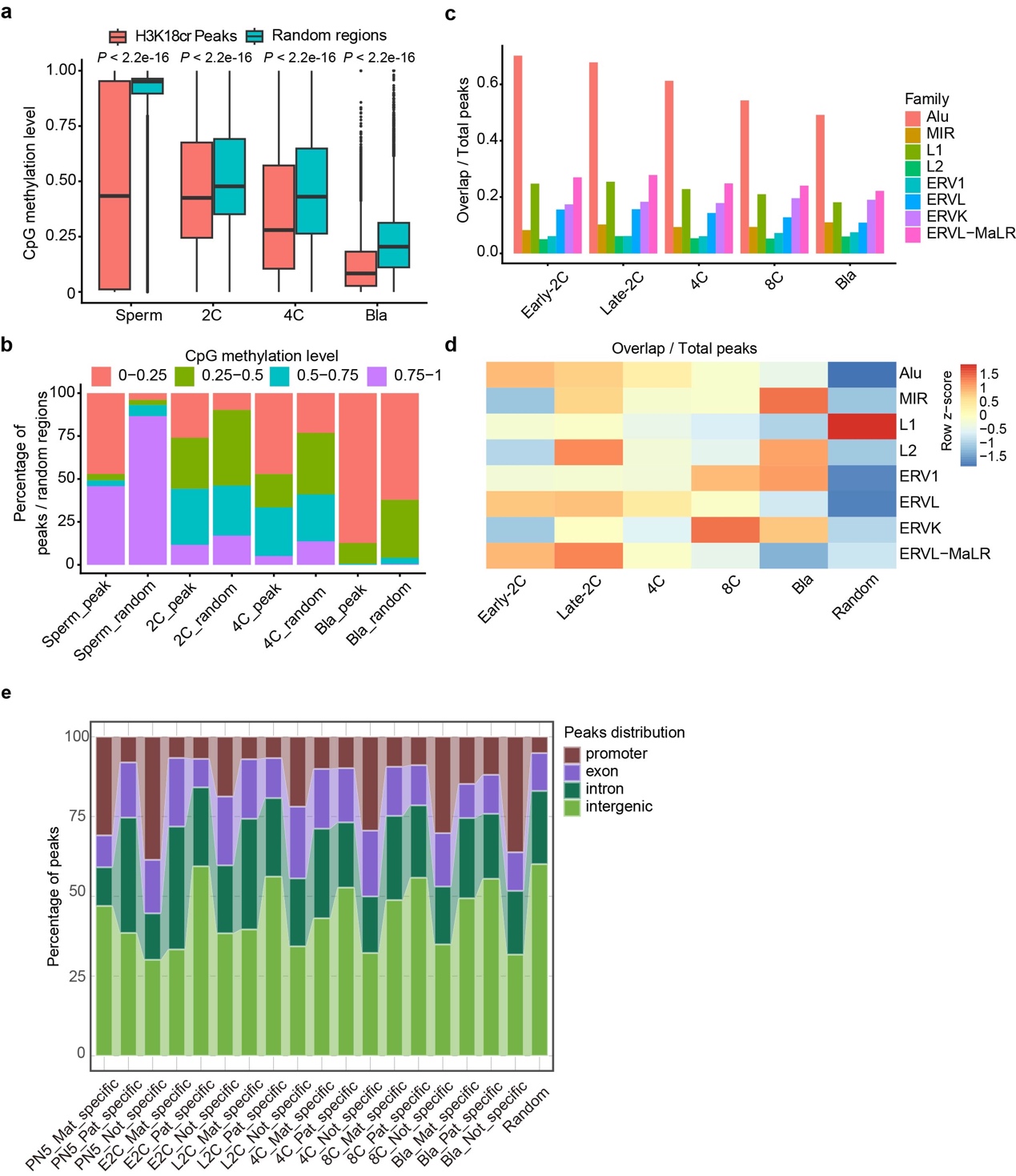


**Figure S5 | Comparison of H3K18cr signal with DNA methylation in mouse gametes and early embryos. a,** Box plot showing the DNA methylation level of H3K18cr peaks and random regions. The Wilcoxon rank-sum test was used. **b,** Percentage distribution of H3K18cr peaks and random regions among four CpG methylation level categories at the indicated stages in mouse gametes and early embryos. CpG methylation levels were grouped as 0–0.25, 0.25–0.5, 0.5–0.75, and 0.75–1. **c,** Bar plot showing the proportion of total H3K18cr peaks overlapping different transposable element families at the indicated stages in mouse early embryos. **d,** Heatmap showing the relative enrichment of different transposable element families in H3K18cr peaks relative to random regions at the indicated stages, displayed as row z-scores. **e,** Bar plot comparing the distribution of parental-specific and non-specific H3K18cr peaks across genomic elements.


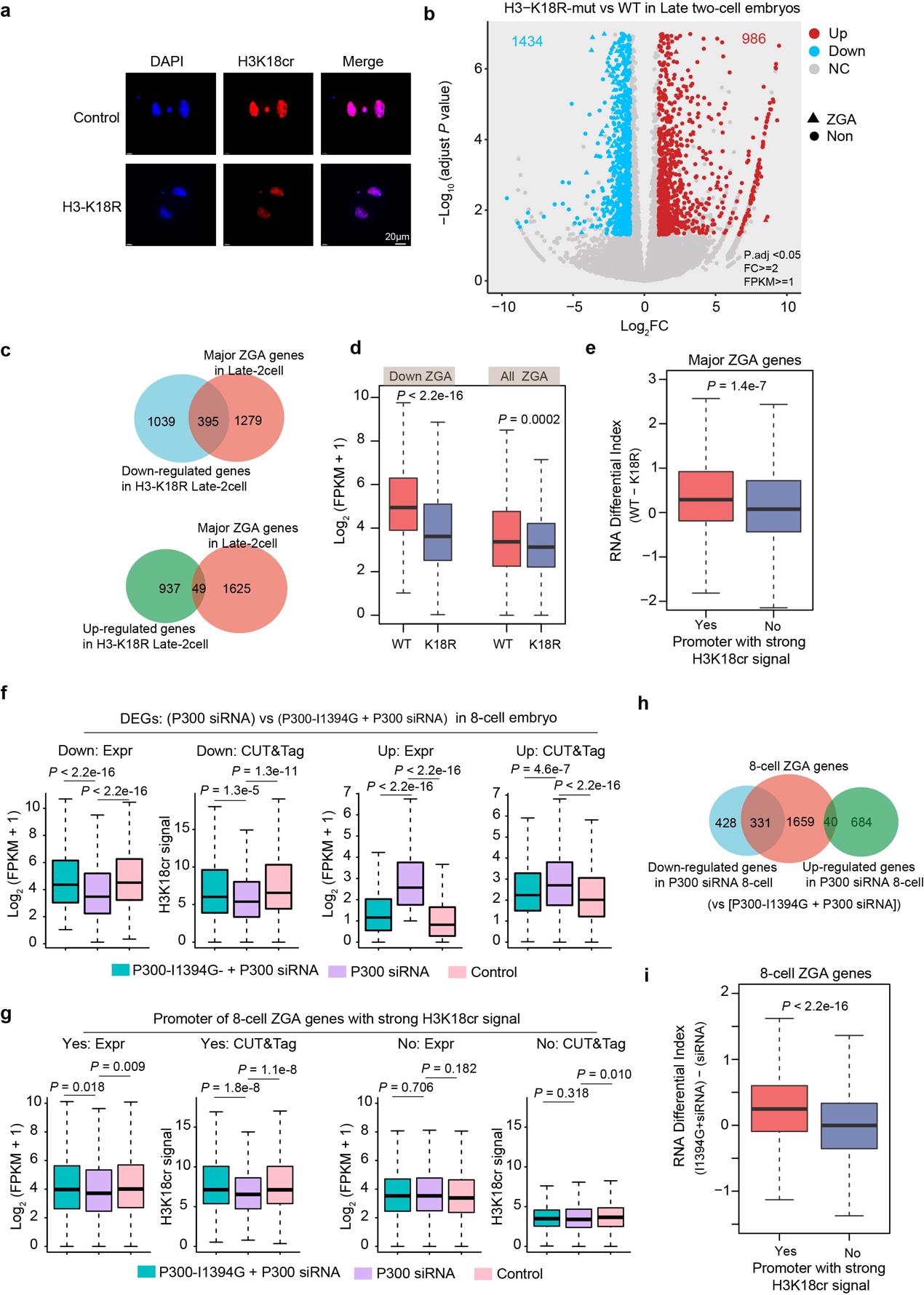


**Figure S6 | Effects of the H3K18R mutation and P300 knockdown on H3K18cr and gene expression. a,** Immunofluorescence staining of H3K18cr in mouse 2-cell embryos showing altered H3K18cr signals after expression of H3K18R mutant mRNA. H3K18R indicates a histone H3 mutant in which lysine 18 is replaced by arginine. Scale bar: 20μm. **b,** Scatter plot showing differentially expressed genes (DEGs) between embryos expressing H3K18R mutant mRNA and those expressing WT (wild-type) H3 mRNA. The cut-offs used to define DEGs are indicated, and ZGA genes are highlighted. The criteria used to define DEGs were shown in the figure. **c,** Venn diagrams showing the overlap between major ZGA genes in late 2-cell embryos and genes that were downregulated or upregulated in H3K18R-expressing late 2-cell embryos. **d,** Box plots comparing the expression of downregulated ZGA genes and all ZGA genes between WT H3- and H3K18R-expressing late 2-cell embryos. Expression levels are shown as log₂(FPKM + 1). Statistical significance was indicated above each comparison. The Wilcoxon rank-sum test was used. **e,** Box plot showing the RNA differential index (WT − K18R) of major ZGA genes grouped by the presence or absence of strong promoter-associated H3K18cr signals. The RNA differential index was calculated as log_2_(FPKM(WT) + 1) − log_2_(FPKM(H3K18R) + 1). The Wilcoxon rank-sum test was used. **f-g,** Box plots comparing gene expression levels (Expr) and promoter-associated H3K18cr CUT&Tag signals (CUT&Tag) among three groups of 8-cell embryos for the indicated DEGs (f) or 8-cell ZGA genes (g) with strong promoter-associated H3K18cr signals. The three groups included control embryos, P300 siRNA embryos, and embryos expressing the P300-I1394G mutant following P300 siRNA treatment (P300-I1394G + P300 siRNA). The P300-I1394G mutant is deficient in histone acetyltransferase (HAT) activity while retaining histone crotonyltransferase (HCT) function. The RNA-seq and CUT&Tag data were obtained from a public dataset (GSE241196). The Wilcoxon rank-sum test was used. **h,** Venn diagrams showing the overlap of 8-cell ZGA genes with genes downregulated or upregulated in P300 siRNA 8-cell embryos compared with P300-I1394G + P300 siRNA 8-cell embryos. **i,** Box plot showing the RNA differential index ([P300-I1394G + P300 siRNA] − [P300 siRNA]) of 8-cell ZGA genes grouped by the presence or absence of strong promoter-associated H3K18cr signals. The Wilcoxon rank-sum test was used.


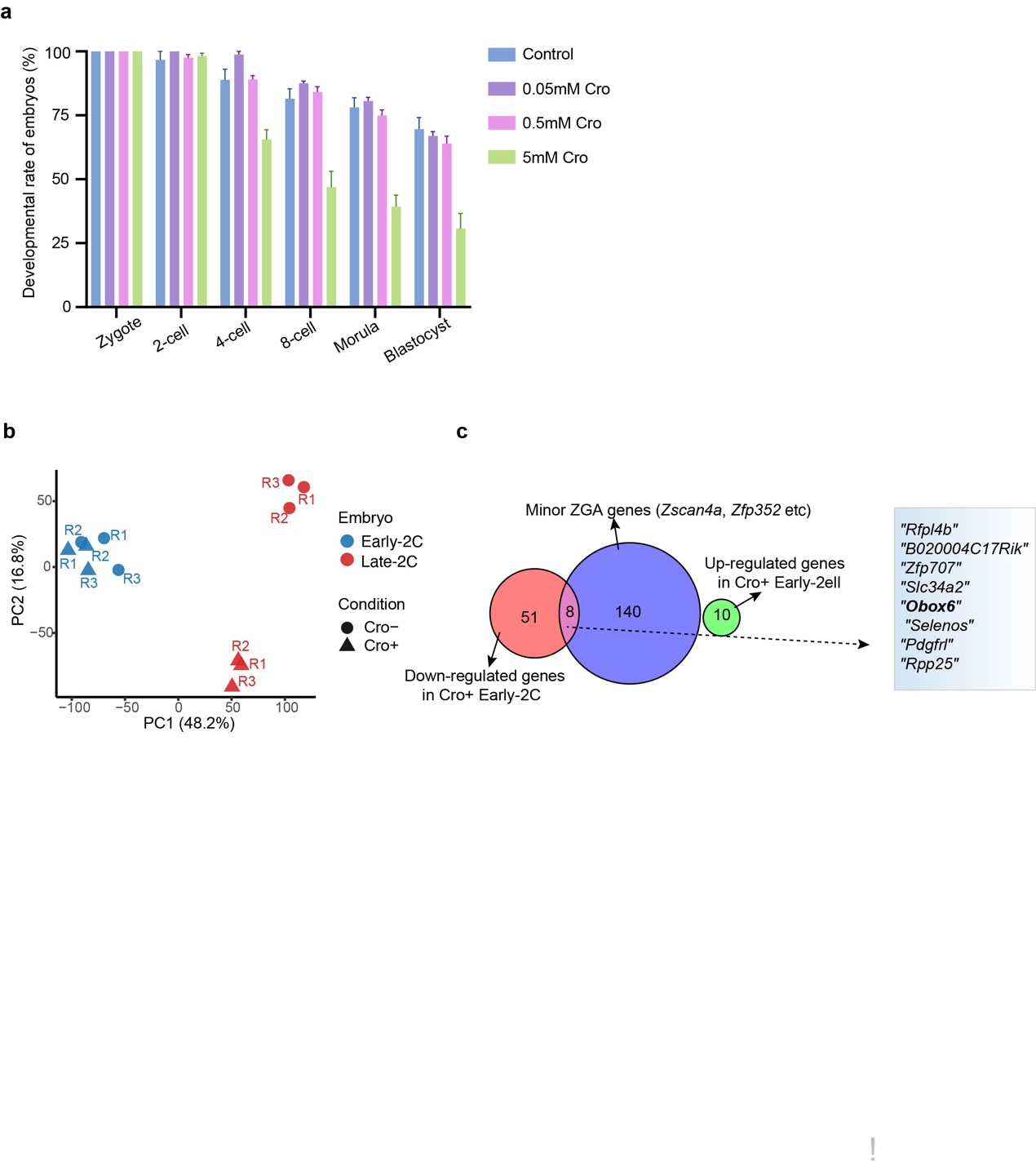


**Figure S7 |** **Effects of treating embryos with crotonic acid on gene expression and minor ZGA.** **a,** Bar plot showing the developmental rates of embryos at the indicated stages following treatment with different concentrations of crotonic acid (Cro) (Control, 0.05 mM, 0.5 mM, and 5 mM). The data were presented as Mean ± SEM of three independent biological replicates. **b,** Principal component analysis (PCA) of crotonic acid–treated and control two-cell embryos based on the RNA-seq data. **c,** Venn diagram shows the number of overlapping genes between genes upregulated or downregulated in early 2-cell embryos after crotonic acid treatment and minor ZGA genes. The blue section on the right shows the downregulated minor ZGA genes.


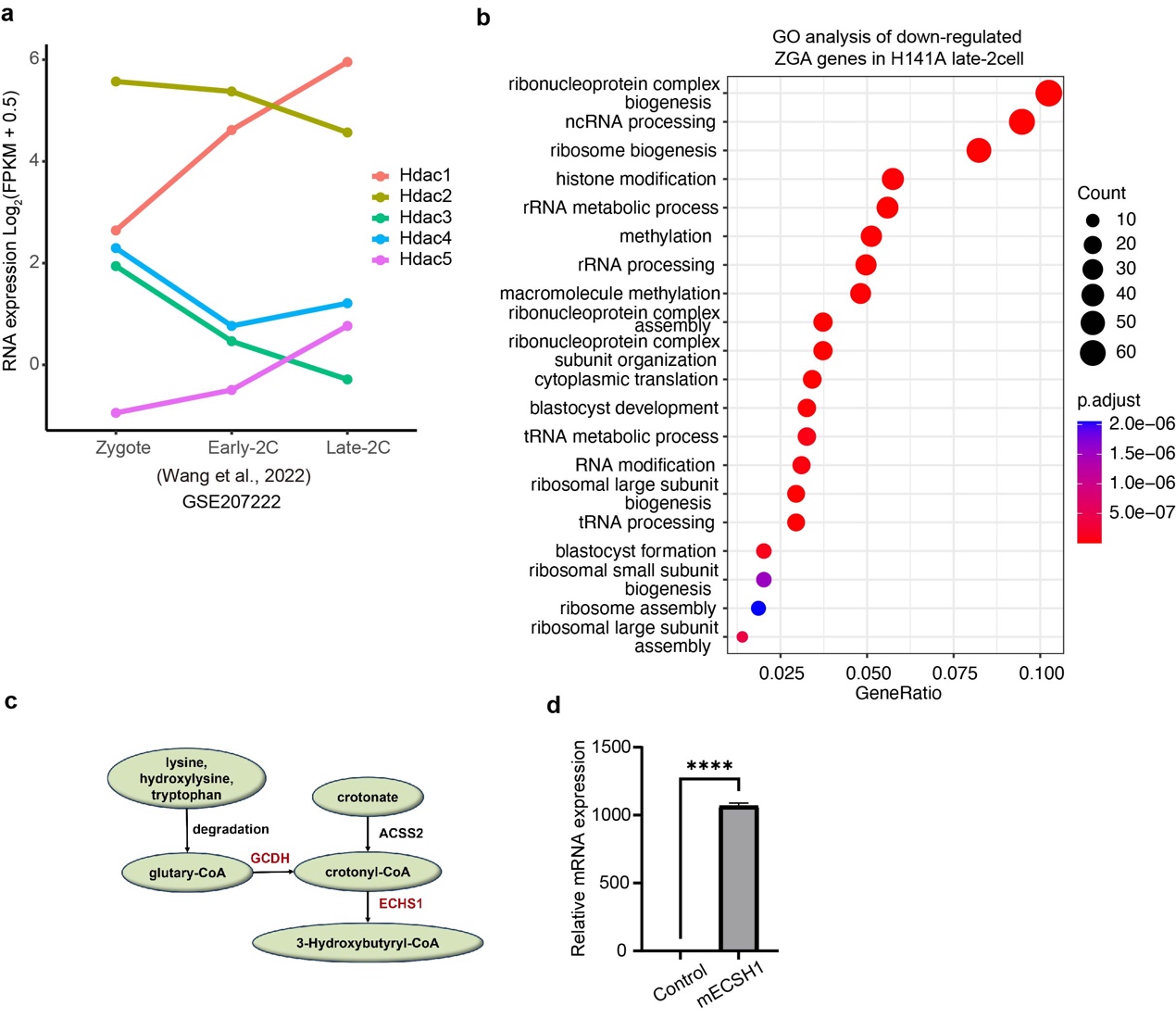


**Figure S8 | Roles of lysine deacetylase activity of HDAC1 H3K18cr reprogramming and ZGA.** **a,** RNA expression of *Hadc1-5* at the indicated stages. **b,** GO analysis of ZGA genes downregulated in late two-cell embryos following HDAC1-H141A overexpression. **c,** Model diagram of key metabolic reactions involved in the generation of crotonyl-CoA. **d,** Validation of overexpression efficiency of mouse *Echs1* (*mEchs1*) mRNA by qRT-PCR in four-cell embryos. The data were presented as Mean ± SEM of three independent biological replicates, and *Gapdh* was used as the reference gene. statistical analysis was performed using two tailed unpaired t-tests. ****P < 0.0001.

**
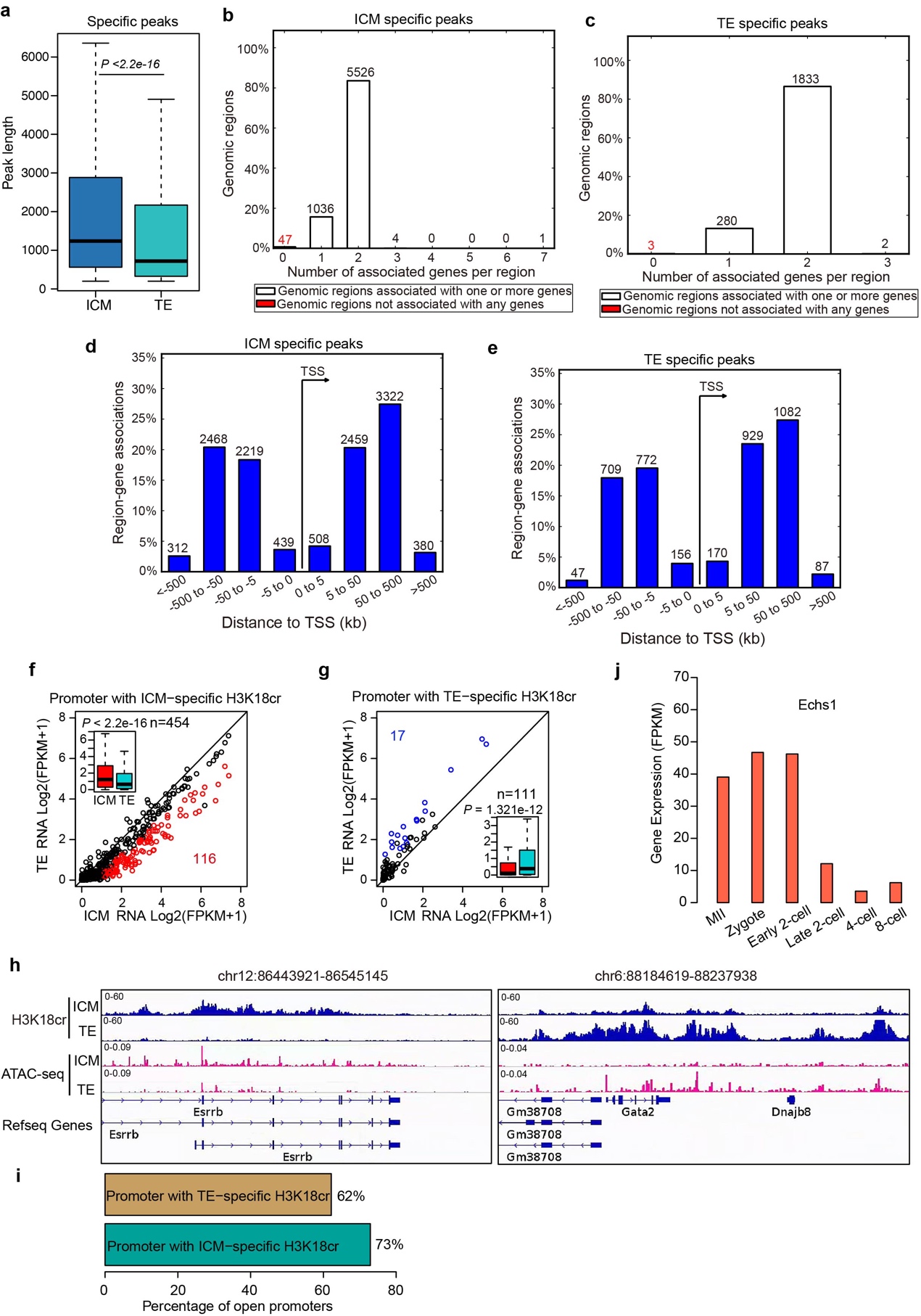
**

**Figure S9 | Features of ICM- and TE-specific H3K18cr peaks and their potential functions. a,** Box plot comparing the length of ICM- and TE-specific H3K18cr peaks. Statistical significance was assessed using the Wilcoxon rank-sum test. **b-c,** Bar charts showing the number or percentage of ICM- (b) and TE- (c) specific H3K18cr peaks (genomic regions) associated with different numbers of genes. **d-e,** The number or percentage of different distance between H3K18cr peaks (genomic regions) and their associated gene transcription start sites (TSS) for ICM-specific (d) and TE-specific (e) H3K18cr peaks. **f,** Scatter plot comparing the RNA expression levels of genes with ICM-specific H3K18cr in promoters between ICM and TE. Red dots represent genes that are downregulated in TE. The Wilcoxon rank-sum test was used. **g,** Scatter plot comparing the RNA expression levels of genes with TE-specific H3K18cr in promoters between ICM and TE. Blue dots represent genes that are upregulated in TE. The Wilcoxon rank-sum test was used. **h,** Genome browser views of H3K18cr and ATAC-seq signals at the *Esrrb* and *Gata2* loci in ICM and TE samples. **i,** Bar plot showing the percentage of open promoters among promoters marked by TE-specific or ICM-specific H3K18cr. ATAC-seq peaks in TE were defined by combining peaks from pre-TE and TE cells in the single-cell NanoATAC-seq2 dataset. **j,** Bar chart showing expression levels of *Echs1* genes in mouse oocytes and preimplantation embryos.

**Legends for the supplementary tables**

**Table S1: The sample information of mouse gametes and embryos used in ULI-NChIP-seq and Smart-seq2.**

**Table S2: The number of peaks with different length.**

**Table S3: GO analysis and ZGA genes with promoters covered by H3K18cr domains that are downregulated in HDAC1-H141A mutant embryo**
